# Integrated multi-omic approach reveals the effect of a *Graminaceae*-derived biostimulant and its lighter fraction on salt-stressed lettuce plants

**DOI:** 10.1038/s41598-024-61576-4

**Published:** 2024-05-10

**Authors:** Sonia Monterisi, Leilei Zhang, Pascual Garcia-Perez, Monica Yorlady Alzate Zuluaga, Michele Ciriello, Christophe El-Nakhel, Valentina Buffagni, Mariateresa Cardarelli, Giuseppe Colla, Youssef Rouphael, Stefano Cesco, Luigi Lucini, Youry Pii

**Affiliations:** 1 Faculty of Agricultural, Environmental and Food Sciences, Free University of Bozen/Bolzano, 39100 Bolzano, Italy; 2 Department for Sustainable Food Process, Università Cattolica del Sacro Cuore, Piacenza, Italy; 3 Department of Agricultural Sciences, University of Naples Federico II, 80055 Portici, Italy; 4 Department of Agriculture and Forest Sciences, University of Tuscia, 01100 Viterbo, Italy

**Keywords:** *Lactuca sativa* L., RNA-seq, Untargeted metabolomics, Biostimulant fractionation, Abiotic stress, Plant sciences, Plant physiology, Plant stress responses

## Abstract

Plant biostimulants are widely applied in agriculture for their ability to improve plant fitness. In the present work, the impact of *Graminaceae*-derived protein hydrolysate (P) and its lighter molecular fraction F3 (< 1 kDa) on lettuce plants, subjected to either no salt or high salt conditions, was investigated through the combination of metabolomics and transcriptomics. The results showed that both treatments significantly modulated the transcriptome and metabolome of plants under salinity stress, highlighting an induction of the hormonal response. Nevertheless, P and F3 also displayed several peculiarities. F3 specifically modulated the response to ethylene and MAPK signaling pathway, whereas P treatment induced a down-accumulation of secondary metabolites, albeit genes controlling the biosynthesis of osmoprotectants and antioxidants were up-regulated. Moreover, according with the auxin response modulation, P promoted cell wall biogenesis and plasticity in salt-stressed plants. Notably, our data also outlined an epigenetic control of gene expression induced by P treatment. Contrarily, experimental data are just partially in agreement when not stressed plants, treated with P or F3, were considered. Indeed, the reduced accumulation of secondary metabolites and the analyses of hormone pathways modulation would suggest a preferential allocation of resources towards growth, that is not coherent with the down-regulation of the photosynthetic machinery, the CO_2_ assimilation rate and leaves biomass. In conclusion, our data demonstrate that, although they might activate different mechanisms, both the P and F3 can result in similar benefits, as far as the accumulation of protective osmolytes and the enhanced tolerance to oxidative stress are concerned. Notably, the F3 fraction exhibits slightly greater growth promotion effects under high salt conditions. Most importantly, this research further corroborates that biostimulants’ mode of action is dependent on plants’ physiological status and their composition, underscoring the importance of investigating the bioactivity of the different molecular components to design tailored applications for the agricultural practice.

## Introduction

Soil salinization is one of the major concerns nowadays since it is strongly contributing to the loss of crops productivity. Primary soil salinization is ascribable to wind, rainfall and parent rock weathering, whilst the secondary is related to anthropogenic activities, which induce salt accumulation at the soil level^1^. One out of five irrigated lands are affected by salinity and every year salt accumulation induces the loss of 1.5 million hectares of agriculture lands^2^; such detrimental effects are further promoted and accelerated by the climate change and the use of low-quality water for irrigation^3^. Furthermore, the future perspectives are even less promising since, by the 2050, 50% of world’s arable land is expected to be impaired by salinity^4^.

Vegetable crops are strongly affected by salinity stress^3^, which can negatively impact the morpho-physiological and biochemical functions in a species-specific manner^5^, resulting in nutritional and ion imbalances, osmotic and oxidative stress, damages to cell membranes, proteins and photosynthetic machinery, and decreasing in plant growth and crop productivity^6^. In this context, being sessile organisms, plants had to develop and adapt many complex mechanisms involved in the morpho-physiological, biochemical and anatomical modifications to cope with salt stress^7^.

Different abiotic stresses share several aspects of the biochemical and molecular response pathways^8^. Salinity, in particular, has been shown to induce the modulation of endogenous phytohormones levels and, consequently, the signaling pathways (e.g., MAPK signaling pathway) involved in the downstream changes in roots, leaves and cellular structures^9–13^. Osmolytes including sugars (e.g., glucose, sucrose and fructose), polyols (e.g., sorbitol, mannitol and glycerol) and charged metabolites (e.g., proline, betaine and glycine) are produced to balance the osmotic stress^14,15^. Moreover, the oxidative damage induced by the salinity stress results in the excessive production of reactive oxygen species (ROS)^16^. To amend the negative effects induced by ROS, plants can produce both antioxidant enzymes (e.g., ascorbate peroxidase—APX, catalase—CAT, superoxide dismutase -SOD, monodehydroascorbate reductase—MDHAR) and molecules^17^.

Nevertheless, the adaptive mechanisms evolved by plants could be not sufficient to efficiently face the impairment induced by the salt stress. For this reason, novel sustainable agronomical approaches are required to face this and others abiotic stresses. In this framework, the application of plant biostimulants (PBs) in agriculture is envisaged as a suitable tool. PBs are defined as products originating from organic or inorganic substances and/or microorganisms, that are able to improve plant growth, productivity and mitigate the negative effects of abiotic stresses^18,19^. Furthermore, PBs can be produced starting from food waste and agro-industrial by-products, thus representing an environmental-friendly solution reducing waste disposal and an advanced strategy to improve the circular economy paradigm^20^.

PBs, by definition, is a variegate group of substances and effectors, and, among them, protein hydrolysates (PHs) represent a subgroup of PBs formed by a mixture of polypeptides, oligopeptides and amino acids obtained by the partial hydrolysis of protein sources^21^. Protein source (either vegetal or animal) and the type of hydrolysis (chemical or enzymatic) strongly influence the PHs mixture composition, which, as a consequence, differentially impact the plant physiology and biochemistry^22^.

Different studies have addressed the ability of PHs, obtained by processing the biomass of plants belonging to different botanical families (e.g., *Graminaceae*, *Malvaceae*, *Brassicaceae*, *Fabaceae*) as protein sources, in ameliorating the resistance of crop plants against abiotic stresses^5,23,24^. In this context, vegetal-derived PHs were shown to alleviate salinity stress by triggering different mechanisms, including (i) the stimulation of carbon (C) and nitrogen (N) metabolisms through the regulation of enzymes involved in the TCA-cycle and N-assimilation pathway^25^; (ii) the enhanced synthesis of antioxidant enzymes and metabolites produced by secondary metabolism^26^; (iii) the modulation of phenylpropanoids metabolism^27^; (iv) the increased photosynthetic metabolism through hormone-like activity^28^; (v) the modulation of gene expression of stress-inducible proteins^29^. Interestingly, these pieces of evidence also showed that, depending on the botanical origin and, hence, on the composition, the effect produced by PHs at plant level (e.g., growth, metabolism) could be different.

The contribution of the single PHs components to the biostimulation process in plants is still unclear; however, the complete elucidation of the bioactivity of single PHs components might greatly contribute to the understanding of their mode of action. At present, very limited knowledge has been gathered in this area. A very recent study showed that *Graminaceae*-derived PH and its molecular fractions induced different effects in lettuce plants grown under salinity stress, highlighting that each fraction could trigger peculiar mechanisms^30^. Consistently, Lucini and colleagues^31^ showed that the lightest fraction (0.5–1 kDa) of a *Fabaceae*-derived PH had the highest bioactivity, stimulating the root development in tomato cuttings through an auxin-like mechanism.

Despite increasing evidence, at present a clear understanding about the effects of whole PHs biostimulants and their molecular fractions on plants molecular and biochemical mechanisms is still missing. Therefore, to overcome this limitation in knowledge, the application of a multidisciplinary approach, coupling for instance metabolomic and transcriptomic analyses, might be necessary shed light on the mechanisms underpinning PHs bioactive effects.

Considering these premises and the results obtained by El-Nakhel et al. (2023), the present work was aimed at investigating the impact of a *Graminaceae*-derived PHs and its lighter molecular fraction F3 (< 1 kDa) on soilless-grown lettuce (*Lactuca sativa* L.) plants, subjected to either no salt or high salt conditions. Lettuce is the most widespread leafy vegetable grown worldwide^32^ due to its high nutritional value. Indeed, it is considered a source of minerals, fiber, vitamins and antioxidant compounds^33^, that are able to boost human health by reducing risk of chronic diseases occurrence (e.g., cancer and diabetes)^34^. However, salinity stress may represent a threat for lettuce growth and yield^35^. In detail, the research focused on the exploration of the influence of the PH and its lightest molecular fraction on the molecular reprogramming of lettuce plants through the combination of two omics techniques, metabolomic and transcriptomic approaches, in both optimal and high salt growing conditions.

The results presented in this work could, on one hand, elucidate the molecular effects of biostimulants administration on the vegetable crop, and, on the other hand, provide a support in the selection of the most bio-active PH fraction(s) according to the desired effects on the plant of interest.

## Results

### Shoot dry weight, physiological and biochemical parameters

**Figure 1:**
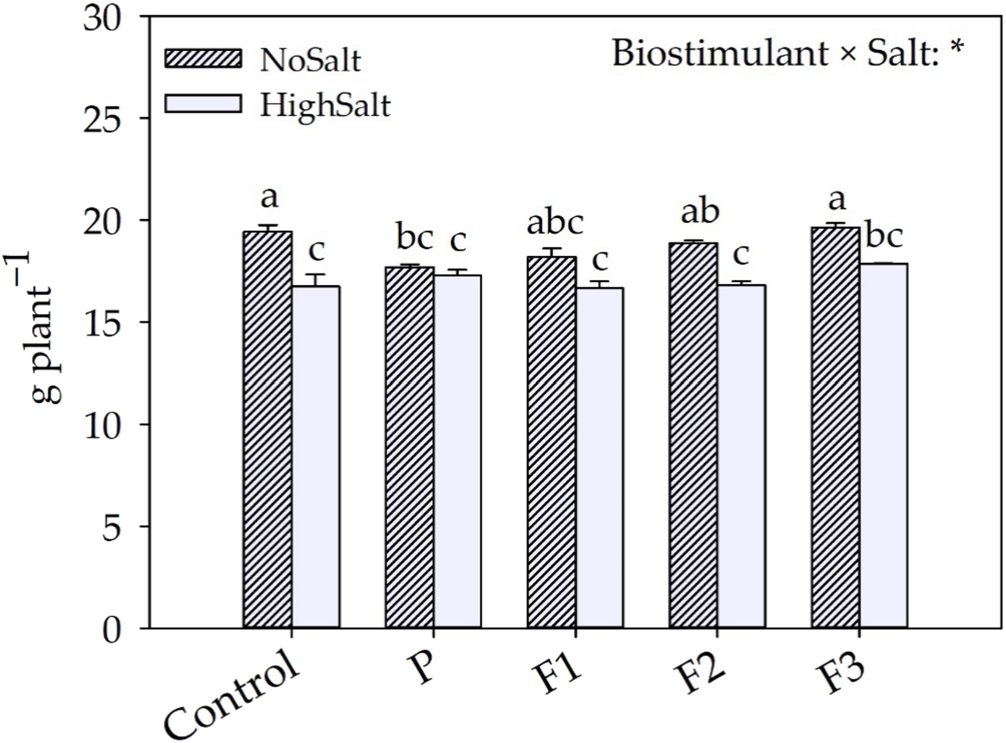
Effects of different salt levels (No Salt and high Salt) and biostimulant fractions (Control, P, F1, F2 and F3) on plant biomass. * denotes a significant effects at *p* ≤ 0.01. Different letters indicate significant differences according to Tukey’s HSD (*p* = 0.05).

As shown in Fig. 1, the shoot dry weight was significantly influenced by the interaction of the two factors evaluated in our experiment (Salt × Biostimulant). Regarding the no salt treatment, the application of P biostimulant resulted in a significant reduction of DW (− 8.9%), whereas all three fractions (F1, F2, and F3) did not induce any significant differences compared to the control. No differences have been induced by biostimulant applications compared to the control in the high salt condition. The physiological and biochemical data of lettuce plants are reported in Table 1. Proline and MDA (biochemical parameters) were significantly affected by the Salt × Biostimulant interaction. Without salt stress, proline did not change under biostimulant treatments, whereas in salt stress conditions all biostimulants determined a significant reduction of the osmolyte (by 35%) compared to control conditions. Lipid peroxidation (MDA) was enhanced for plants subjected to high salt condition and not treated with biostimulants (Control) or subjected to F3 treatment. Same values have been observed for the interaction No Salt × F2. Furthermore, all biostimulant treatments reduced MDA parameter for plants grown in the absence of salt stress. Regardless of biostimulants treatments, plants grown under high salinity conditions recorded the lowest values of all biometric parameters except WUEi. In relation to the average biostimulant effect, the use of fractions of F2 and F3 increased, on average, A_CO2_ by 10.1% compared to the control (Table 1).

**Table 1:** Physiological and biochemical parameters of *Lactuca sativa* L. grown under two levels of salinity and sprayed with a *Graminaceae*-based protein hydrolysate (P) and its three molecular fractions (F1, F2 and F3).

Treatment	A_CO2_	g_s_	E	WUEi	Proline	MDA
	(μmol CO_2_ m^−2^ s^−1^)	(mmol H_2_O m^−2^ s^−1^)	(mmol H_2_O m^−2^ s^−1^)	(μmol CO_2_ mmol H_2_O^-1^)	(mmol 100 g^-1^ FW)	(µM 100 g^-1^ FW)
Salt (S)						
NoSalt	18.44 ± 0.18 a	0.2 ± 0 a	4.17 ± 0.08 a	4.45 ± 0.11 b	18.27 ± 0.53 b	0.96 ± 0.04
HighSalt	17.82 ± 0.31 b	0.19 ± 0.01 b	3.73 ± 0.1 b	4.82 ± 0.13 a	44.1 ± 2.68 a	0.98 ± 0.03
t-test	*	*	***	*	***	*n.s*
Biostimulant (B)						
Control	17.05 ± 0.54 b	0.18 ± 0.01	3.8 ± 0.23	4.57 ± 0.29	41.24 ± 9.53 a	1.06 ± 0.03 a
P	18.04 ± 0.3 ab	0.2 ± 0.01	3.97 ± 0.15	4.57 ± 0.15	28.58 ± 4.82 b	0.89 ± 0.01 c
F1	18.03 ± 0.35 ab	0.19 ± 0.01	4 ± 0.17	4.54 ± 0.19	27.63 ± 4.64 b	0.94 ± 0.04 bc
F2	19.02 ± 0.23 a	0.2 ± 0.01	4.04 ± 0.12	4.74 ± 0.2	30.15 ± 5.25 b	1 ± 0.09 ab
F3	18.53 ± 0.17 a	0.19 ± 0.01	3.93 ± 0.18	4.76 ± 0.23	28.31 ± 5.47 b	0.97 ± 0.05 b
	***	*n.s*	*n.s*	*n.s*	***	***
S × B						
NoSalt*Control	17.83 ± 0.57	0.2 ± 0	4.21 ± 0.07	4.24 ± 0.18	21.18 ± 0.74 c	1.03 ± 0.03 bc
NoSalt *P	18.22 ± 0.51	0.21 ± 0.01	4.24 ± 0.17	4.3 ± 0.06	17.88 ± 0.95 c	0.86 ± 0.01 d
NoSalt *F1	18.68 ± 0.22	0.2 ± 0	4.25 ± 0.11	4.4 ± 0.11	17.56 ± 0.44 c	0.86 ± 0.01 d
NoSalt *F2	18.97 ± 0.29	0.2 ± 0.01	4 ± 0.21	4.78 ± 0.34	18.6 ± 0.95 c	1.19 ± 0.03 a
NoSalt *F3	18.51 ± 0.25	0.2 ± 0.01	4.14 ± 0.31	4.53 ± 0.41	16.1 ± 0.56 c	0.85 ± 0.01 d
HighSalt*Control	16.27 ± 0.71	0.16 ± 0.01	3.39 ± 0.3	4.89 ± 0.53	61.3 ± 7.18 a	1.1 ± 0.03 ab
HighSalt *P	17.86 ± 0.39	0.19 ± 0.01	3.7 ± 0.08	4.83 ± 0.19	39.28 ± 0.95 b	0.91 ± 0.02 cd
HighSalt *F1	17.38 ± 0.35	0.19 ± 0.01	3.75 ± 0.26	4.68 ± 0.38	37.71 ± 2.4 b	1.02 ± 0.01 bc
HighSalt *F2	19.07 ± 0.43	0.21 ± 0.01	4.07 ± 0.17	4.71 ± 0.28	41.7 ± 1.96 b	0.8 ± 0.05 d
HighSalt *F3	18.55 ± 0.28	0.19 ± 0.01	3.73 ± 0.16	5 ± 0.21	40.52 ± 0.11 b	1.08 ± 0.02 ab
	*n.s*	*n.s*	*n.s*	*n.s*	**	***

Control corresponds to lettuce plants sprayed with water. All data are expressed as mean ± standard error, n = 3.

A_CO2_ = net assimilation rate; g_s_ = stomatal conductance; E = transpiration; WUEi = Instantaneous water use efficiency; MDA = malondialdehyde.

### RNA-seq analysis on lettuce plants

The RNA-seq analysis was performed aiming at investigating how the biostimulant P and its fraction F3, which showed the most promising agronomic performances, could modulate the lettuce transcriptome in normal and high salt conditions.

The transcript sequencing produced 49,289,894, 51,150,056 and 44,464,926 raw reads for control, P and F3 plants in high salt conditions, respectively. For no salt treated plants, instead, the raw reads number were 47,171,164, 43,719,009 and 50,332,875 for control, P and F3 plants, respectively (Supplementary Table S1). Reads filtering resulted in 7.1 G of clean bases on average for all the treatments in both salt conditions. Furthermore, at least the 90% of the clean bases had a Q Phred higher than 30 (Supplementary Table S1).

On average 90% of the reads were aligned to a unique position in the reference genome (Supplementary Table S1).

**Figure 2:**
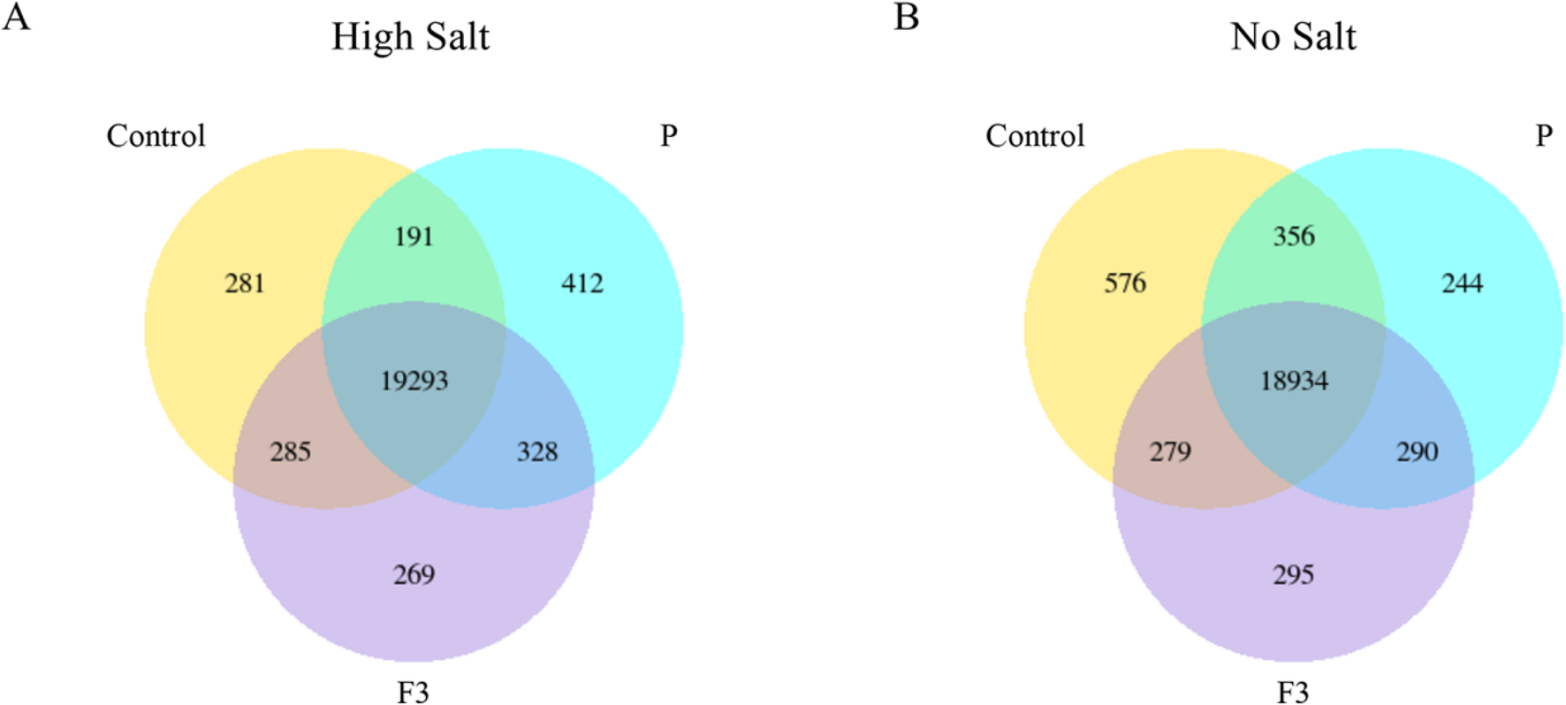
Venn diagrams reporting the number of DEGs in lettuce plants grown in either High (**A**) or No (**B**) salt conditions and either not treated (Control) or treated with the biostimulant P or its fraction F3.

In salinity stress conditions, lettuce plants treated with biostimulant P had 412 genes uniquely expressed, whereas those treated with the fraction F3 had 269 genes uniquely expressed (Fig. 2A). The control plants, instead, had 281 genes uniquely expressed most likely driven by the stress condition (Fig. 2A). On the other hand, the three treatments investigated shared 19,293 differentially expressed genes (DEGs). In no salt condition, the number of DEGs shared among the three treatments considered was 18,934 (Fig. 2B). Nonetheless, a decrease in the number of genes uniquely expressed in plants treated with P (244 genes) was detected, whilst an increase was assessed in control plants (576 genes). Peculiar DEGs detected in plants treated with the PH fraction F3 were approximately in the same amount in both high and no salt conditions (269 and 295, respectively) (Fig. 2).

The statistical analysis, carried out with DESeq2 Software^36^ using the thresholds *p* value ≤ 0.05 and log2|FoldChange(FC)|≥ 0.0, allowed the evaluation of the significant up- and down- regulated genes for each treatment in both high and no salt conditions.

**Figure 3:**
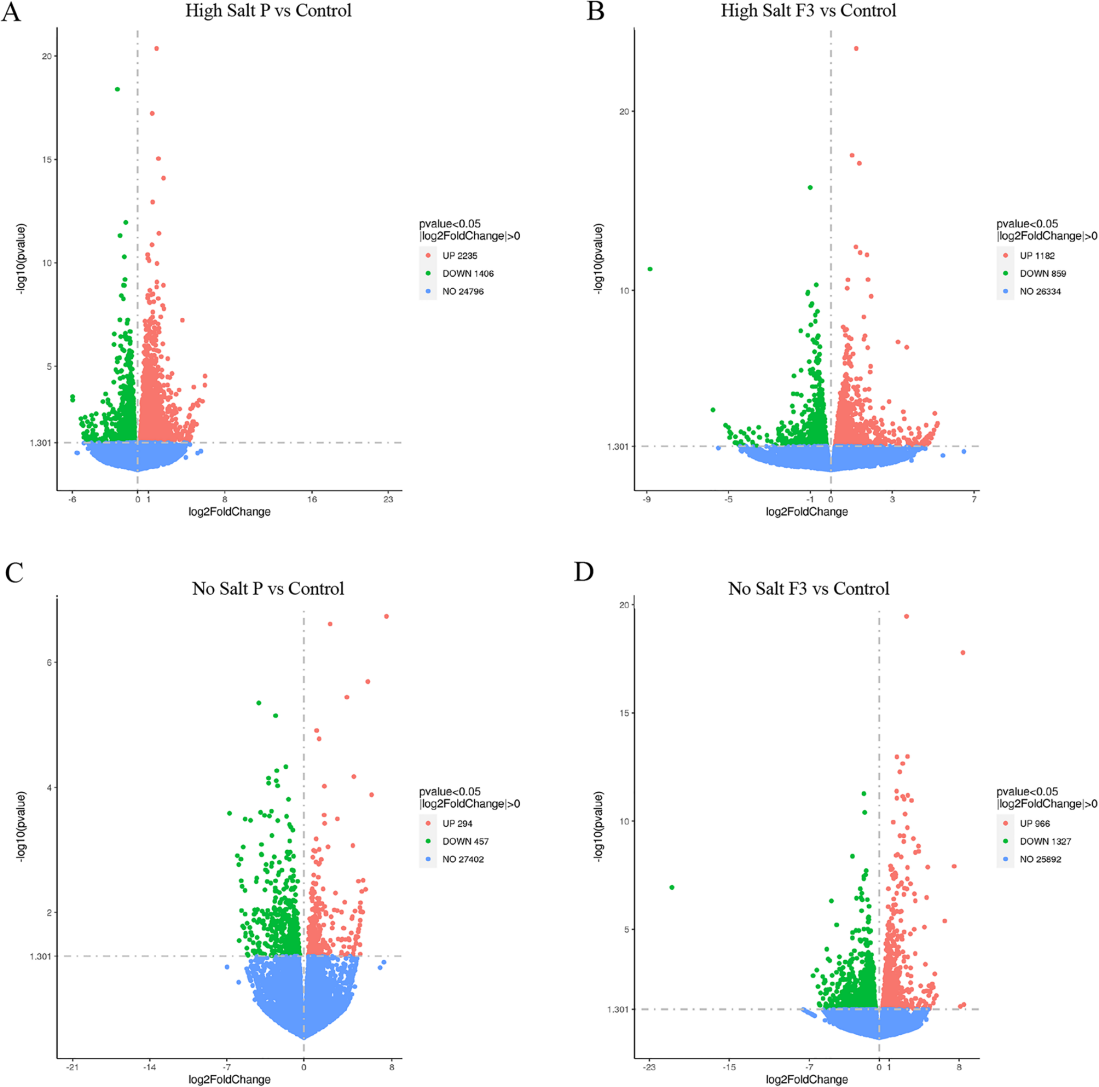
Volcano Plot of DEGs detected in lettuce plants exposed to different salinity conditions and biostimulant treatments. (**A**) High Salt, P versus Control, (**B**) High Salt, F3 versus Control, (**C**) No Salt, P versus Control, (**D**) No Salt, F3 versus Control. The DEGs were selected using a threshold of |Log2FC|> 0 and *P* < 0.05. UP, upregulated; DOWN, downregulated; NO, not changed.

In high salt condition, the treatment with biostimulant P significantly up- and down-regulated 2235 and 1406 genes, respectively, whereas the tratment with the F3 fraction induced the significant up-regulation of 1182 genes and the down-regulation of 859 genes (Fig. 3A and B). Plants grown in no salt condition and treated with biostimulant P showed a decrease in the number of significantly modulated genes, being 294 and 457 genes up-and down-regulated, respectively (Fig. 3C). On the contrary, the treatment with the fraction F3 on plants grown in no salt conditions induced the modulation of a similar number of genes as observed in salt-stressed plants, showing 1327 down-regulated genes and 966 up-regulated genes (Fig. 3D).

### Functional analysis of differentially expressed genes

**Figure 4:**
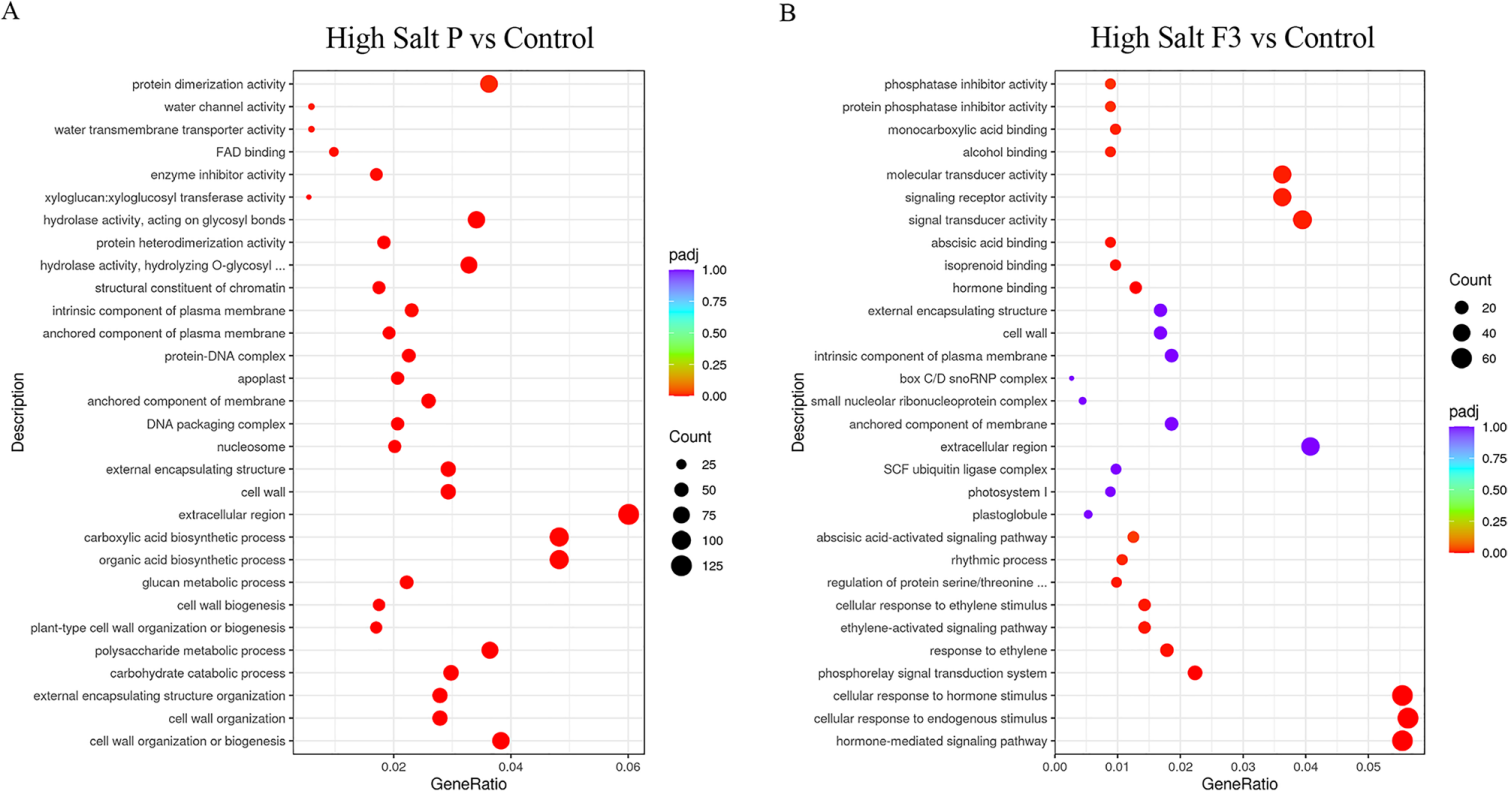
Gene Ontology Enrichment analyses for plants grown in high salt condition. (**A**) GO Enrichment analyses in plants treated with biostimulant P with respect to Control plants. (**B**). GO Enrichment analyses in plants treated with biostimulant fraction F3 with respect to Control plants. The top 30 ranked GO terms according to gene count, adjusted *p* value and gene ratio. ‘Gene count’ is the number of genes enriched in a GO term. ‘Gene ratio’ is the percentage of total DEGs in the given GO term. Plots have been generated by using clusterProfiler R package v3.8.1.

**Figure 5:**
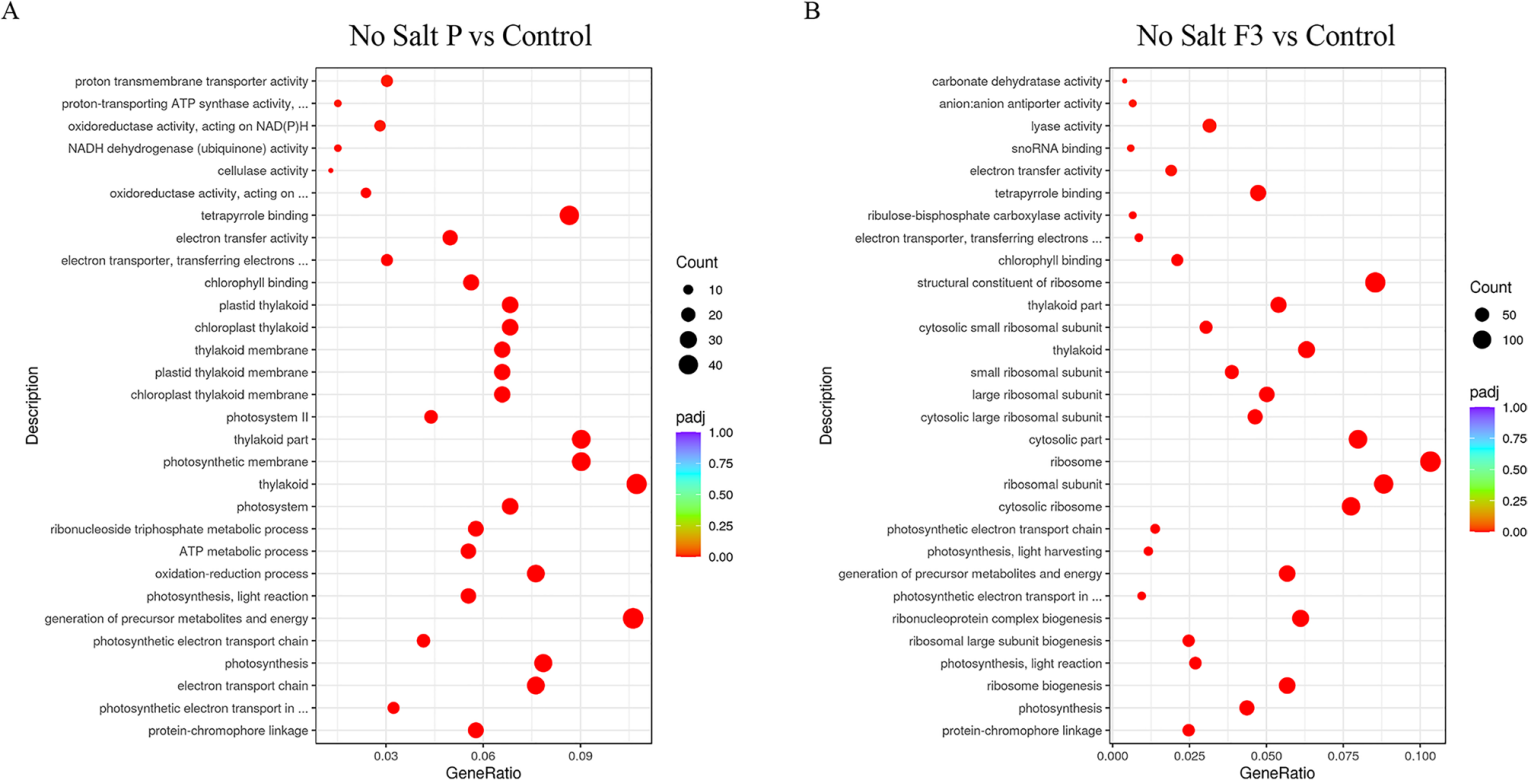
Gene Ontology Enrichment analyses for plants grown in no salt condition. (**A**) GO Enrichment analyses in plants treated with biostimulant P with respect to Control plants. (**B**). GO Enrichment analyses in plants treated with biostimulant fraction F3 with respect to Control plants. The top 30 ranked GO terms according to gene count, adjusted *p* value and gene ratio. ‘Gene count’ is the number of genes enriched in a GO term. ‘Gene ratio’ is the percentage of total DEGs in the given GO term. Plots have been generated by using clusterProfiler R package v3.8.1.

The DEGs identified in the two salt conditions and following the treatments with the biostimulant P and its fraction F3 were further classified according to Gene Ontology (GO) terms, i.e., cellular component (CC), molecular function (MF) and biological processes (BP). The thirty most significant GO terms (padj < 0.05), affected by the treatment with either biostimulant P or its fraction F3, in both high and no salt conditions, were selected and reported in Figs. 4 and 5. The genes reported in the following sections were selected among those showing a |log2FC|≥ 2 and belonging to the MF and BP terms of GO.

#### High salt conditions

In high salt regime the biostimulant P significantly affected several genes involved in the BP terms *cell wall organization or biogenesis*, *cell wall organization*, *external encapsulating structure organization, plant-type cell wall organization or biogenesis* and *cell wall biogenesis*. Within these terms, eight genes showed a significant up-regulation and one was significantly down-regulated (Fig. 4 and Supplementary Table S2). One of the up-regulated and the down-regulated genes encoded for pectinesterases (*LSAT_5X137700* and *LSAT_3X127861*), whilst the remaining up-regulated genes encoded for a COBRA-like protein 7 (*LSAT_6X115101*), a TED6_ZINVI Protein tracheary element differentiation-related 6 (*LSAT_9X107661*), a glucuronoxylan 4-O methyltransferase 1 (*LSAT_1X25320*), an expansin-A6 (*LSAT_5X170161*), a probable xyloglucan endotransglucosylase/hydrolase protein 32 (*LSAT_6X24740*), a cellulose synthase-like protein E1 (*LSAT_5X2221*) and a callose synthase 7 (*LSAT_3X34861*).

Within the BP terms *carbohydrate catabolic process*, *polysaccharide metabolic process* and *glucan metabolic process,* nine genes were up-regulated and one gene was down-regulated (Supplementary Table S2). Among the up-regulated genes, six of them (*LSAT_5X137700, LSAT_3X127861, LSAT_1X25320, LSAT_6X24740, LSAT_5X2221*, *LSAT_3X34861*) have already been mentioned above. Beside these, the endoglucanases 17 (*LSAT_8X163780* and *LSAT_9X5920*), endoglucanase 6 (*LSAT_6X84061*) and a gene encoding for callose synthase 7 (*LSAT_3X35021*) were also up-regulated.

The *organic acid biosynthetic process* and *carboxylic acid biosynthetic process* BP terms were represented by seven and one genes significantly up-regulated and down-regulated, respectively (Supplementary Table S2). The up-regulated genes encoded for a putative amino-acid acetyltransferase NAGS2 (*LSAT_4X161500*), a L-gulonolactone oxidase 3 (*LSAT_5X33021*), a protein breast cancer susceptibility 1 homolog (*LSAT_7X32301*), a putative L-gluconolactone oxidase 6 (*LSAT_4X41641*), a putative linoleate 9S-lipoxygenase 5 (*LSAT_9X86780*), a nodulin-related protein 1 (*LSAT_3X56161*) and a 3-ketoacyl-CoA synthase 19 (*LSAT_2X116461*); the down-regulated one encoded for an alpha-dioxygenase PIOX (*LSAT_2X92600*).

The MF terms significantly affected by the P treatment included the *structural constituent of chromatin, protein heterodimerization activity* and *protein dimerization activity,* whereby twenty genes were significantly up-regulated (Supplementary Table S2). Ten of these encoded for histone H3.2 (*LSAT_5X89880*, *LSAT_6X99500*, *LSAT_1X9380*, *LSAT_7X10540*, *LSAT_6X99580*, *LSAT_6X41341*, *LSAT_6X81961*, *LSAT_6X99480*, *LSAT_1X67140*, *LSAT_6X99380*), one gene for histone H2A.1 (*LSAT_9X87241*), four genes encoded for histone H4 (*LSAT_9X65201*, *LSAT_1X129540*, *LSAT_1X129381*, *LSAT_1X129540*), one for histone H2B.9 (*LSAT_8X22980*), one for histone H2B.3 (*LSAT_8X17800*) and one for a putative histone H2A.3 (*LSAT_0X7041*). Within the term *protein dimerization activity,* two genes encoding for transcription factor bHLH94 (*LSAT_6X106080*) and bHLH25 (*LSAT_2X81721*) were up-regulated.

The treatment with biostimulant P also affected the MF terms *hydrolase activity hydrolyzing O-glycosyl compounds*, *hydrolase activity acting on glycosyl bonds* and *xyloglucan:xyloglucosyl transferase activity,* by inducing the modulation of ten genes, nine up-regulated and one down-regulated (Supplementary Table S2). Among the up-regulated, two genes encoded for endoglucanase 17 (*LSAT_8X163780* and *LSAT_9X5920*), one for a nod factor hydrolase protein 1 (*LSAT_8X59300*), one for an endoglucanase 6 (*LSAT_6X84061*), two for beta-fructofuranosidase soluble isoenzyme I (*LSAT_9X59600* and *LSAT_2X125041*), one for a probable xyloglucan endotransglucosylase/hydrolase protein 32 (*LSAT_6X24740*), one for glucan endo-1,3-beta-glucosidase 12 (*LSAT_7X95080*) and one for polygalacturonase QRT3 (*LSAT_2X60921*). On the other hand, the down-regulated gene encoded for a glucan endo-1,3-beta-glucosidase 13 (*LSAT_9X93180*).

Furthermore, the MF term *enzyme inhibitor activity* was affected by the treatment with the biostimulant P displaying the up-regulation of four genes (Supplementary Table S2), encoding for a cysteine proteinase inhibitor B (*LSAT_1X69140*), a pectinesterase (*LSAT_5X137700*), a pectinesterase inhibitor 9 (*LSAT_3X2521*) and a putative membrane-associated kinase regulator 3 (*LSAT_2X111341*). Concerning the MF term *FAD binding*, P-treated plants showed the up-regulation of three genes encoding for L-gulonolactone oxidase 3 (*LSAT_5X33021*), a putative L-gulonolactone oxidase 6 (*LSAT_4X41641*) and a Tetrahydrocannabinolic acid synthase (*LSAT_1X56720*), and the down-regulation of a gene encoding for berberine bridge enzyme-like 8 (*LSAT_3X107581*).

Finally, the treatment with biostimulant P also affected the MF terms *water transmembrane transporter activity* and *water channel activity*, within which genes encoding for aquaporins were both up-regulated, i.e., putative aquaporin PIP2-8 (*LSAT_8X123201*), and down-regulated, i.e., putative aquaporin TIP-type RB7-5A (*LSAT_6X91781*) (Supplementary Table S2).

The F3 fraction also induced significant changes in the gene expression of treated plants in high salt condition compared to the control lettuce. The BP terms *hormone-mediated signaling pathway*, *cellular response to endogenous stimulus* and *cellular response to hormone stimulus* were significantly affected by the treatment with the up-regulation of two genes (Supplementary Table S2) encoding for ethylene responsive transcription factor 1B (*LSAT_2X107800*) and histidine-containing phosphotransfer protein 4 (*LSAT_4X97481*). The same genes were also detected within other BP terms significantly impacted by the F3 treatment, *phosphorelay signal transduction system*, *response to ethylene*, *ethylene-activated signaling pathway* and *cellular response to ethylene stimulus.*

Other BP and MF terms were affected by the F3 treatment, yet not showing statistical significance (Supplementary Table S2). These terms include *abscisic acid-activated signaling pathway*, *cellular response to abscisic acid stimulus*, *cellular response to alcohol*, *regulation of protein serine/threonine phosphatase activity*, *rhythmic process*, *hormone binding*, *isoprenoid binding*, *abscisic acid binding*, *alcohol binding*, *monocarboxylic acid binding*, *protein phosphatase inhibitor activity* and *phosphatase inhibitor activity*. Interestingly, within the MF terms *signal transducer activity*, *signaling receptor activity* and *molecular transducer activity*, the up-regulation of the same gene encoding for histidine-containing phosphotransfer protein 4 (*LSAT_4X97481*) and the down-regulation of a gene encoding for mitogen-activated protein kinase homolog MMK2 (*LSAT_3X138401*) were detected (Supplementary Table S2).

#### No salt conditions

GO enrichment analysis was also performed on plants grown in no salt conditions treated with P biostimulant and compared with untreated lettuce plants. The BP terms significantly affected by the treatment included *protein-chromophore linkage*, *photosynthetic electron transport in photosystem II*, *electron transport chain*, *photosynthesis*, *photosynthetic electron transport chain*, *generation of precursor metabolites and energy*, *photosynthesis light reaction* and *oxidation–reduction process* (Fig. 5A). Whitin these terms, thirty-nine genes significantly changed their expression (Supplementary Table S2). One gene encoding for 2,3-bisphosphoglycerate-dependent phosphoglycerate mutase 1 (*LSAT_6X102220*) was up-regulated, whereas thirty eight genes were down-regulated and included three genes for photosystem II D1 protein (*LSAT_8X133980*, *LSAT_3X104200*, *LSAT_4X51841*), one for photosystem I iron-sulphur centre (*LSAT_1X14180*), cytochrome c oxidase subunit 2 (*LSAT_6X95140*) and subunit 3 (*LSAT_8X111680*), delta-9-acyl-lipid desaturase 1 (*LSAT_1X33441*), eight genes for photosystem I P700 chlorophyll a apoprotein A2 (*LSAT_4X147760*), A1-like (*LSAT_1X88121*), A1 (*LSAT_4X147740*, *LSAT_4X102100*, *LSAT_5X152620*, *LSAT_1X110681*) and A2-like (*LSAT_5X152640*, *LSAT_1X110661*), six for photosystem II D2 protein (*LSAT_8X1941*, *LSAT_0X46221*, *LSAT_1X88080*, *LSAT_7X5761*, *LSAT_4X58161*, *LSAT_3X99281*), one for cytochrome b (*LSAT_0X44400*), four for photosystem II CP43 reaction centre protein (*LSAT_2X50780*, *LSAT_2X34800*, *LSAT_4X147781*, *LSAT_4X128060*), one for photosystem II CP47 reaction centre protein (*LSAT_8X11001*), four for NADH-ubiquinone oxidoreductase chain 1 (*LSAT_4X66041*), chain 2 (*LSAT_0X38140*) and chain 4 (*LSAT_1X88121*, *LSAT_8X46780*), three for NAD(P)H-quinone oxidoreductase subunit 1 (*LSAT_4X118121*) and subunit H (*LSAT_4X33860*, *LSAT_9X49240*), one for ATP synthase subunit beta (*LSAT_3X99261*) and three for ribulose bisphosphate carboxylase large chain (*LSAT_7X14980*, *LSAT_2X70541*) and large chain-like (*LSAT_4X102701*).

The BP terms *ATP metabolic process* and *ribonucleoside triphosphate metabolic process* were also significantly affected by the treatment with P biostimulant in no salt conditions, thereby showing eighteen genes significantly modulated. Seven of these genes have been previously mentioned (*LSAT_6X102220*, *LSAT_8X111680*, *LSAT_6X95140*, *LSAT_1X33441*, *LSAT_3X99261*, *LSAT_8X46780*) (Supplementary Table S2), whilst the further eleven genes were significantly down-regulated. Among these, eight genes encoded for ATP synthase subunit a (*LSAT_8X19261*), alpha (*LSAT_1X2921*, *LSAT_8X1961*, *LSAT_1X88060*), b (*LSAT_3X117960*, *LSAT_1X38680*) and c (*LSAT_2X1621*, *LSAT_1X38660*), two genes encoded for NADH-ubiquinone oxidoreductase chain 2 (*LSAT_0X38140*) and chain 4 (*LSAT_4X39621*) and one encoded for ATP synthase protein MI25 (*LSAT_0X35060*) (Supplementary Table S2).

Concerning the MF category, *chlorophyll binding*, *electron transporter transferring electrons within the cyclic electron transport pathway of photosynthesis activity*, *electron transfer activity* and *tetrapyrrole binding* terms were significantly affected by the treatment with P biostimulant (Supplementary Table S2). Beside several genes already mentioned in the previous GO terms (*LSAT_8X133980*, *LSAT_3X104200*, *LSAT_4X51841, LSAT_4X147760*, *LSAT_1X88121*, *LSAT_4X147740*, *LSAT_4X102100*, *LSAT_5X152620*, *LSAT_1X110681*, *LSAT_8X1941*, *LSAT_0X46221*, *LSAT_1X88080*, *LSAT_7X5761*, *LSAT_4X58161*, *LSAT_3X99281, LSAT_2X50780*, *LSAT_2X34800*, *LSAT_4X147781*, *LSAT_4X128060, LSAT_8X11001, LSAT_1X14180, LSAT_6X95140, LSAT_8X111680* and *LSAT_0X44400*), three genes encoding for Photosystem II D2 protein (*LSAT_5X16100*), alkane hydroxylase MAH1 (*LSAT_9X92240*) and cytochrome P450 94C1 (*LSAT_1X48460*) were found to be down-regulated, whilst four encoding for cytochrome P450 704C1 (*LSAT_9X19341*), cytochrome P450 81Q32 (*LSAT_1X23761*), premnaspirodiene oxygenase (*LSAT_7X82580*) and ent-kaurenoic acid oxidase 1 (*LSAT_4X41921*) resulted up-regulated (Supplementary Table S2).

Moreover, the MF terms *oxidoreductase activity acting on NAD(P)H, quinone or similar compound as acceptor*, *NADH dehydrogenase (ubiquinone) activity* and *oxidoreductase activity acting on NAD(P)H* were significantly affected by P treatment in no salt conditions, featuring nine genes differentially expressed. Six of these genes were already present in other terms previously described (*LSAT_4X66041*, *LSAT_0X38140*, *LSAT_8X46780*, *LSAT_4X118121*, *LSAT_4X33860*, *LSAT_9X49240*), whereas two genes encoding for NADH-ubiquinone oxidoreductase chain 4 (*LSAT_4X39621*) and NADH dehydrogenase [ubiquinone] iron-sulfur protein 2 (*LSAT_7X67841*) resulted down-regulated and one gene encoding for glutaredoxin-C1 (*LSAT_8X116281*) was up-regulated (Supplementary Table S2). Furthermore, only one gene encoding for endoglucanase 11 (*LSAT_7X92741*), belonging to the MF term *cellulase activity*, was significantly down-regulated (Supplementary Table S2).

Finally, the MF terms *proton-transporting ATP synthase activity rotational mechanism* and *proton transmembrane transporter activity* were significantly impacted by the biostimulant treatment in no salt condition with thirteen genes differentially expressed (*LSAT_8X111680*, *LSAT_2X1621*, *LSAT_8X19261*, *LSAT_1X2921*, *LSAT_8X1961*, *LSAT_3X117960*, *LSAT_1X38660*, *LSAT_1X38680*, *LSAT_1X88060*, *LSAT_3X99261*, *LSAT_6X95140*, *LSAT_4X128060*, *LSAT_0X35060*), already described above (Supplementary Table S2).

The same investigation was carried out in lettuce plants grown in no salt conditions and treated with the biostimulant fraction F3 (Fig. 5B). The F3 treatment affected several BP terms, in particular *protein-chromophore linkage*, *photosynthesis light reaction*, *photosynthetic electron transport in photosystem II*, *generation of precursor metabolites and energy*, *photosynthesis light harvesting*, *photosynthetic electron transport chain*, *electron transport chain* and *photosynthesis light harvesting in photosystem I*, encompassing thirty-five genes significantly down-regulated (Supplementary Table S2). These genes encoded for cytochrome b (*LSAT_0X44400*), photosystem I iron-sulphur centre (*LSAT_1X14180*), photosystem II D2 protein (*LSAT_8X1941*, *LSAT_7X5761*, *LSAT_1X88080*, *LSAT_4X58161*, *LSAT_0X46221*, *LSAT_3X99281*), photosystem I P700 chlorophyll a apoprotein A1 (*LSAT_4X147740*), A1-like (*LSAT_1X88121*) and A2 (*LSAT_4X147760*), NAD(P)H-quinone oxidoreductase subunit H (*LSAT_9X49240*, *LSAT_4X33860*) and chain 4 (*LSAT_1X14200*), photosystem II CP43 reaction centre protein (*LSAT_2X34800*, *LSAT_2X50780*, *LSAT_4X147781*, *LSAT_4X128060*), NADH-ubiquinone oxidoreductase chain 2 (*LSAT_0X38140*) chain 1 (*LSAT_4X66041*) and chain 4 (*LSAT_3X105380*, *LSAT_4X39621*, *LSAT_8X46780*), cytochrome c oxidase subunit 2 (*LSAT_6X95140*) and subunit 3 (*LSAT_8X111680*), glyoxysomal malate synthase (*LSAT_5X13501*), delta-9 acyl-lipid desaturase 1 (*LSAT_1X33441*), photosystem II D1 protein (*LSAT_8X133980, LSAT_4X51841*), NADH dehydrogenase [ubiquinone] iron-sulphur protein 2 (*LSAT_7X25900*), photosystem II CP47 reaction centre protein (*LSAT_8X11001*), ATP synthase subunit beta (*LSAT_1X98641*, *LSAT_3X99261*, *LSAT_4X102720*) and cytochrome b559 subunit alpha (*LSAT_2X43901*).

The BP term *photosynthesis* included twenty-five genes significantly down-regulated (Supplementary Table S2). Among these, nineteen were also included in the BP terms previously described (*LSAT_1X14180*, *LSAT_8X1941*, *LSAT_7X5761*, *LSAT_1X88080*, *LSAT_4X58161*, *LSAT_0X46221*, *LSAT_3X99281, LSAT_4X147740*, *LSAT_1X88121*, *LSAT_4X147760, LSAT_2X34800*, *LSAT_2X50780*, *LSAT_4X147781*, *LSAT_4X128060, LSAT_8X133980, LSAT_4X51841, LSAT_4X33860, LSAT_8X11001, LSAT_2X43901*), whereas the remaining six genes resulted specific to this term. One of these encodes for photosystem I P700 chlorophyll a apoprotein A2-like (*LSAT_5X152640*), while the other five encode for ribulose bisphosphate carboxylase large chain (*LSAT_7X14980*, *LSAT_4X102701*, *LSAT_3X110220*, *LSAT_2X70541*, *LSAT_2X89801*) (Supplementary Table S2).

The treatment with F3 significantly affected other BP terms, like *ribosome biogenesis*, *ribosomal large subunit biogenesis* and *ribonucleoprotein complex biogenesis*, causing the down-regulation of two genes encoding for 40S ribosomal protein S27-2 (*LSAT_8X83181*) and 30S ribosomal protein S11 (*LSAT_6X69061*), and the up-regulation of one gene encoding for endoribonuclease YBEY (*LSAT_6X7781*) (Supplementary Table S2).

Concerning the MF category, the *structural constituent of ribosome* term was significantly impacted by treatment with the biostimulant fraction F3, displaying the down-regulation of seven genes (Supplementary Table S2). Two of these have been previously mentioned in the *ribosome biogenesis*, *ribosomal large subunit biogenesis* and *ribonucleoprotein complex biogenesis* terms (*LSAT_8X83181*, *LSAT_6X69061*), whereas the other five genes encoded for ATP synthase subunit a (*LSAT_8X19261*), 30S ribosomal protein S3 (*LSAT_3X22620*) and S2 (*LSAT_5X26601*), 50S ribosomal protein L2 (*LSAT_3X113260*) and 60S ribosomal protein L5 (*LSAT_7X25860*).

Furthermore, the MF terms *chlorophyll binding* and *electron transporter transferring electrons within the cyclic electron transport pathway of photosynthesis activity* were affected by the F3 treatment with the significant down-regulation of sixteen genes (Supplementary Table S2), already detected within previously described BP terms (*LSAT_8X1941*, *LSAT_7X5761*, *LSAT_1X88080*, *LSAT_4X58161*, *LSAT_0X46221*, *LSAT_3X99281*, *LSAT_4X147740*, *LSAT_1X88121*, *LSAT_4X147760*, *LSAT_2X34800*, *LSAT_2X50780*, *LSAT_4X147781*, *LSAT_4X128060*, *LSAT_8X133980, LSAT_4X51841*, *LSAT_8X11001*).

The MF terms *Lyase activity*, *ribulose-bisphosphate carboxylase activity* and *carbonate dehydratase activity* have also been affected by the treatment with F3 fraction, showing the significant down-regulation of nine genes (Supplementary Table S2). Five of them were already present in the *photosynthesis* term (*LSAT_7X14980*, *LSAT_4X102701*, *LSAT_3X110220*, *LSAT_2X70541*, *LSAT_2X89801*), whereas the other four encoded for bifunctional 3-dehydroquinate dehydratase/shikimate dehydrogenase (*LSAT_4X140321*), carbonic anhydrase Nec1 (*LSAT_1X51421*), 4-hydroxy-tetrahydrodipicolinate synthase (*LSAT_4X56041*) and alpha carbonic anhydrase 7 (*LSAT_1X63840*).

Moreover, the MF term *tetrapyrrole binding* was significantly impacted by the treatment with F3 fraction, displaying twenty-eight genes differentially expressed. Among these, twenty-five genes were down-regulated (Supplementary Table S2) and seventeen of them (*LSAT_8X1941*, *LSAT_7X5761*, *LSAT_1X88080*, *LSAT_4X58161*, *LSAT_0X46221*, *LSAT_3X99281*, *LSAT_4X147740*, *LSAT_1X88121*, *LSAT_4X147760*, *LSAT_2X34800*, *LSAT_2X50780*, *LSAT_4X147781*, *LSAT_4X128060*, *LSAT_8X133980, LSAT_4X51841, LSAT_8X11001*, *LSAT_2X43901*) have already been described within other GO terms. On the other hand, eight genes resulted specific to this term and encoded for lignin-forming anionic peroxidase (*LSAT_4X21960*), probable cytochrome c biosynthesis protein (*LSAT_5X16100*), cytochrome P450 CYP82D47 (*LSAT_4X120300*) and 76C1 (*LSAT_6X51200*), peroxidase N1 (*LSAT_9X85400*), beta-amyrin 28-monooxygenase (*LSAT_5X81721*), geraniol 8-hydroxylase (*LSAT_3X2800*) and cationic peroxidase 1 (*LSAT_5X150921*). Three genes belonging to this term, instead, were significantly up-regulated and encoded for nematode resistance protein-like HSPRO2 (*LSAT_2X121400*), xanthotoxin 5-hydroxylase CYP82C4 (*LSAT_1X19741*) and cytochrome P450 86A1 (*LSAT_8X71141*).

The last three MF terms that have been significantly influenced by the treatment with the biostimulant fraction F3 in lettuce plants grown in no salt conditions were *electron transfer activity*, *snoRNA binding* and *anion:anion antiporter activity.*

Within the *electron transfer activity* term, eighteen genes (*LSAT_0X44400*, *LSAT_1X14180*, *LSAT_8X1941*, *LSAT_7X5761*, *LSAT_1X88080*, *LSAT_4X58161*, *LSAT_0X46221*, *LSAT_3X99281*, *LSAT_4X147740*, *LSAT_1X88121*, *LSAT_4X147760*, *LSAT_2X34800*, *LSAT_2X50780*, *LSAT_4X147781*, *LSAT_6X95140*, *LSAT_8X111680*, *LSAT_8X133980*, *LSAT_4X51841*) were significantly down-regulated and previously detected in others BP terms. Lastly, the *anion:anion antiporter activity* term was represented by a single gene encoding for ADP, ATP carrier protein 1 (*LSAT_1X66200*), which resulted significantly down-regulated (Supplementary Table S2).

#### KEGG metabolic pathway analysis

The effects of the two biostimulants treatments, i.e., the P protein hydrolysate and its fraction F3, on lettuce plants grown in either high or no salt conditions, were further deepened by investigating the metabolic pathway significantly affected (padj < 0.05) by DEGs using the KEGG collection^37–39^.

**Figure 6:**
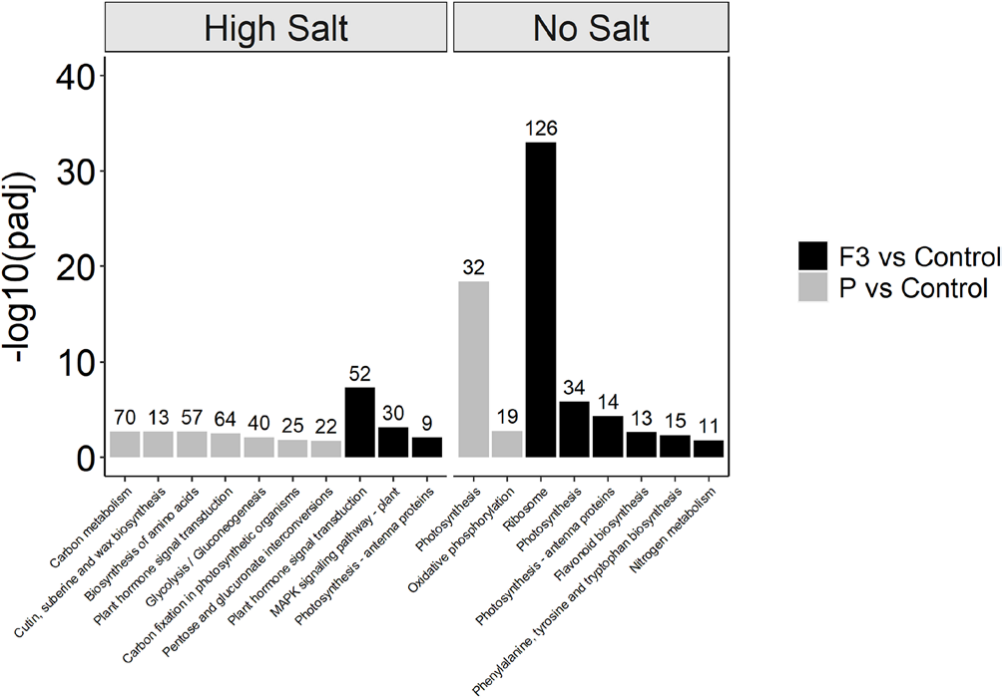
Statistically enriched KEGG pathways in the comparison groups. Significantly enriched KEGG pathways are determined on the base of *p* adjust < 0.05. The number on top of each bar represent the number of DEG significantly affected in the indicated pathway.

In high salt conditions, the application of biostimulant P significantly affected seven KEGG pathway with a -log_10_(padj) ranging between 1.7 and 2.7. These influenced pathways, listed from the most to the less significant, were *carbon metabolism*, *cutin, suberine and wax biosynthesis*, *biosynthesis of amino acids*, *plant hormone signal transduction*, *glycolysis/gluconeogenesis*, *carbon fixation in photosynthetic organisms* and *pentose and glucuronate interconversions*. These pathways feature a number of DEGs ranging from 70 to 22, respectively (Fig. 6).

The application of biostimulant fraction F3, instead, significantly affected three pathways in high salt condition with a − log_10_(padj) ranging between 2.1 and 7.3. The most significantly affected pathway was *plant hormone signal transduction*, also detected in plants treated with biostimulant P, followed by *MAPK signaling pathway—plant* and *photosynthesis—antenna proteins*. These pathways were characterized by the modulation of 52, 30 and 9 DEGs, respectively (Fig. 6).

The treatment with P biostimulant significantly affected two KEGG pathways in lettuce plants cultivated with no salt addition, namely *photosynthesis* and *oxidative phosphorylation*, with a − log_10_(padj) of 18.4 and 2.8, respectively. Within these two pathways, 32 and 19 genes resulted differentially modulated (Fig. 6).

The application of biostimulant fraction F3 lettuce plants grown in no salt conditions, instead, significantly affected six pathways, i.e., *ribosome*, *photosynthesis*, *photosynthesis—antenna proteins*, *flavonoid biosynthesis*, *phenylalanine, tyrosine and tryptophan biosynthesis* and *nitrogen metabolism*, with a -log_10_(padj) ranging from 1.8 to 33 (Fig. 6).

Interestingly, both *photosynthesis* and *photosynthesis-antenna proteins* pathways have also been detected in plants treated with biostimulant P grown in no salt and high salt conditions, respectively. However, in this case, these featured a higher number of DEGs (34 and 14, respectively).

The *plant hormone signal transduction* KEGG pathway (Supplementary Fig. S1) resulted affected in lettuce plants grown in high salt conditions treated with either the biostimulant P or its fraction F3, albeit this latter showed a higher statistical significance (− log_10_(padj) = 7.3). However, in both treatments the up-regulation of genes involved in the auxin signal transduction was observed. In particular, *TIR1* genes, the auxin receptor factors, which works with Aux/IAAs as co-receptor complex, as well as of others primary auxin responsive genes (i.e., *SAUR* and *GH3*), was up-regulated. Differently, only plants treated with biostimulant P and grown in high salt conditions showed the up-regulation of *Aux/IAA* genes, which act as transcriptional repressors of early auxin-responsive gene expression.

For the cytokinin response pathway, following the treatment with either biostimulant P or its fraction F3, the up-regulation of type-A *ARRs* genes, which encodes for negative regulators of cytokinin signalling, was detected in plants grown in high salt conditions (Supplementary Fig. S1). On the other hand, only plants treated with the fraction F3 showed the concomitant up-regulation of the *AHP* genes, classified as positive regulators of cytokinin signalling pathway.

Concerning the abscisic acid pathway, lettuce plants grown in high salt conditions and treated with both the biostimulant P and its fraction F3 showed the up-regulation of *PYR/PYL* genes and the down-regulation of ABA responsive genes *ABF*. Differently, the *PP2C* gene was specifically downregulated in plants treated with P biostimulant (Supplementary Fig. S1).

Ethylene signal transduction pathways showed instead an up-regulation of *ETR*s and *ERF1/2* genes following the treatment with both biostimulant P and its fraction F3 in plants exposed to high salt conditions, whilst P treatment specifically caused the down-regulation of *SIMKK* genes. Furthermore, both treatments caused the up-regulation *JAZ* gene involved in the jasmonic acid signalling pathway^40^. On the other hand, also biostimulant-specific effects were observed. In fact, P treatment specifically affected the brassinosteroids signalling pathway with the up-regulation of *BSK*, *BZR1/2*, *TCH4* and *CYCD3* genes, whilst the treatment with F3 fraction down-regulated the genes encoding for *TGA* transcription factors involved in the salicylic acid signalling pathway.

In high salt regime, the treatment with biostimulant P significantly impacted the *carbon metabolism* up-regulating 54 genes over the 70 detected (Data not shown) and the *biosynthesis of amino acids*, up-regulating the majority of genes detected within this pathway (49 over the 57 in total) (Data not shown).

On the other hand, when considering the specific effects of the treatment with biostimulant fraction F3 in plants grown in high salt conditions, the modulation of the *MAPK signalling pathway- plant* was detected, with the down-regulation of *MAPK4* gene involved in the pathogen response, and the *MAPK 4/6* genes involved in the cold and salt stress response. Consistently, it was also detected the up-regulation of *PYR/PYL* genes involved in the salt/drought/osmotic stress response, as well as, of genes involved in the ethylene response (Supplementary Fig. S2).

Independently from the growing conditions, the treatment with the biostimulant fraction F3 showed a significant impact on *photosynthesis -antenna proteins* (Supplementary Fig. S3). It caused the down-regulation of genes encoding for light-harvesting chlorophyll protein complex (LHC) *a4*, *b1*, *b3* and *b6,* in both high and no salt regime, and of LHC *b2* and *b5*, specifically in no salt conditions (Supplementary Fig. S3).

Focusing on plants grown in no salt conditions, the treatment with either the biostimulant P or its fraction F3 significantly affected the *photosynthesis* pathway, causing the down-regulation of genes encoding different proteins involved in the Photosystem II (*psbA*, *psbD*, *psbC*, *psbB*, *psbE* and *psb28*), Photosystem I (*psaA*, *psaB* and *psaC*) and F-type ATPase (*beta*, *alpha*, *c*, *a* and *b*) functioning (Supplementary Fig. S4).

Notably, the treatment with biostimulant fraction F3 strongly affected the *ribosome* pathway in plants grown in no salt conditions (− log_10_(padj) = 33.02), inducing the down-regulation of 126 genes encoding for ribosomal proteins (Supplementary Fig. S5).

### Metabolomics analysis

**Figure 7:**
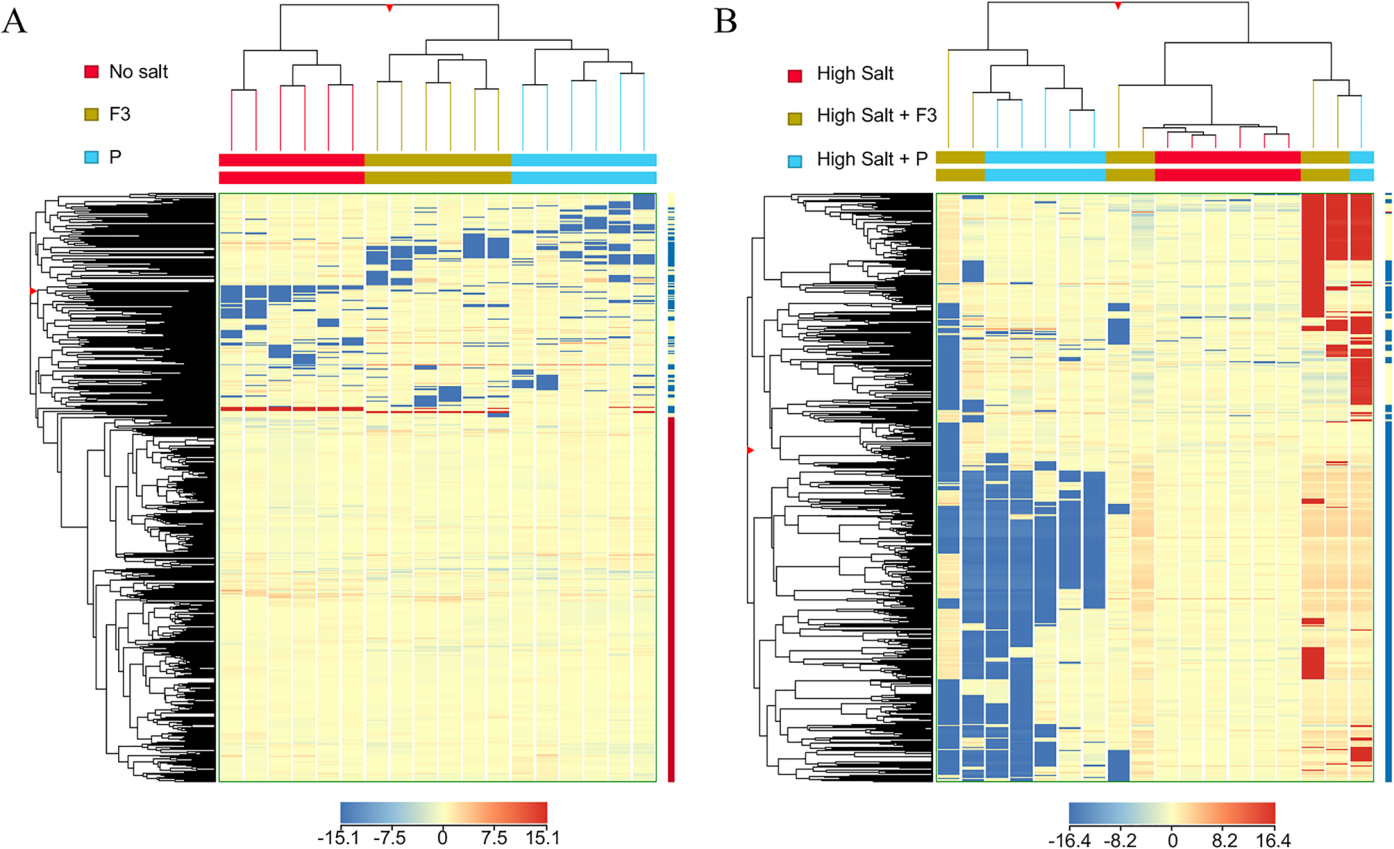
Hierarchical clustering of lettuce plants treated with biostimulant P and its F3 fraction under no salt (**A**) or high salt (**B**) conditions. Plots have been generated by Mass Profiler Professional B.14.

Untargeted metabolomics analysis performed by UHPLC-QTOF-MS provided an overall 3050 putatively annotated metabolites (Supplementary Table S3). The unsupervised hierarchical cluster analysis was carried out on the normalized dataset, and separated by stressed and unstressed groups (Fig. 7), highlighting a clear metabolic modulation produced by biostimulants application in both experimental groups. The effect of biostimulant metabolic modulation was most relevant under salinity stress conditions revealing their potential functionality under abiotic stress.

**Figure 8:**
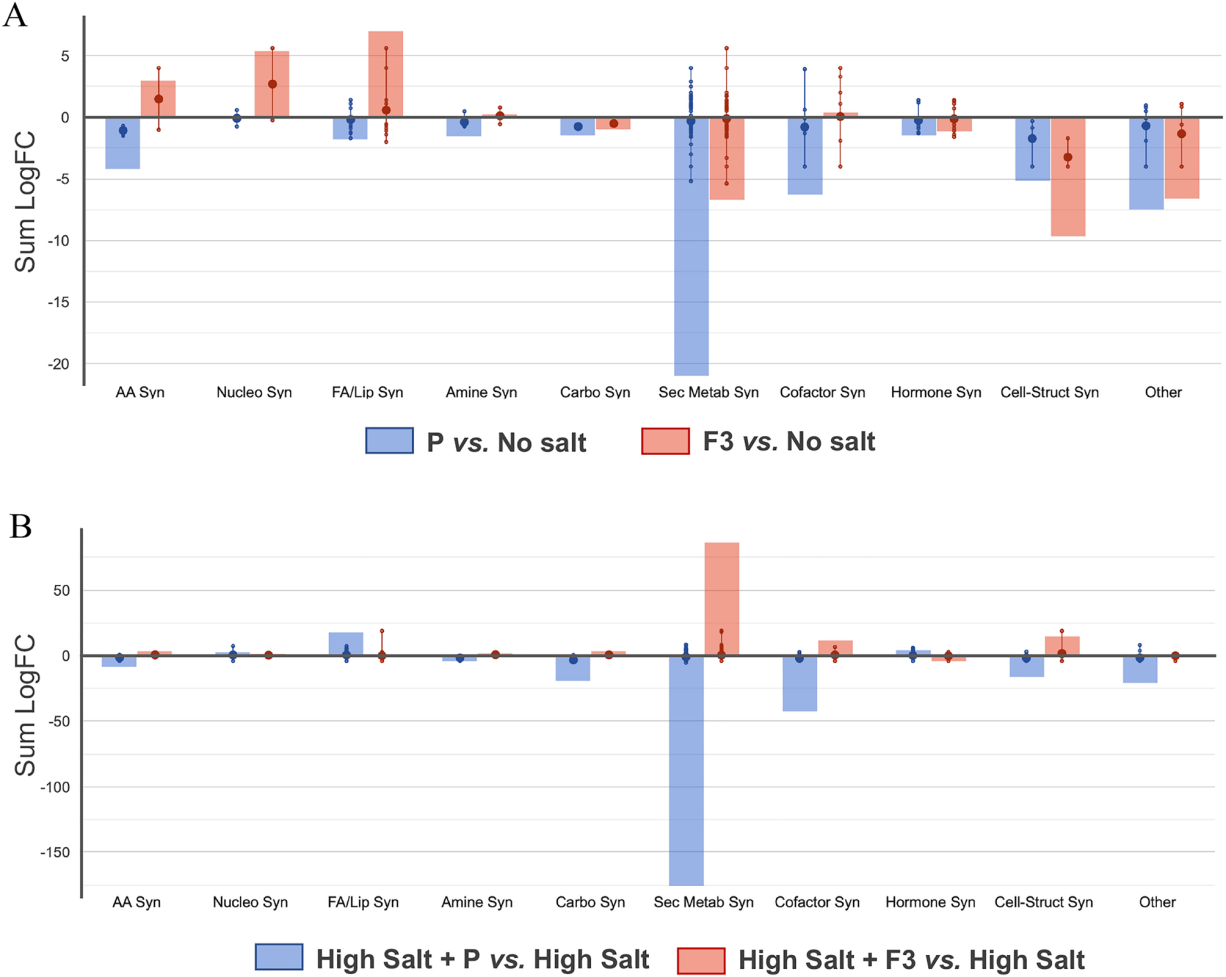
PlantCyc pathways analysis of lettuces’ biosynthetic processes treated with biostimulant P and its F3 fraction under no salt (**A**) or high salt (**B**) coditions. Differential metabolites were used through Volcano plot analysis (*p* < 0.05, fold-change > 1.5). Compounds categories are reported on the x-axis, while cumulative fold changes are given on the y-axis. Abbreviations: AA = amino acids; Nucelo = nucleotides; FA/Lip = fatty acids and lipids; Carbo = carbohydrates; Sec Metab = secondary metabolites; Cell = Struct = cell structures.

The main metabolic pathways affected by biostimulants in both stressed and unstressed conditions were assessed by the PlantCyc tool pathways analysis, using metabolites that resulted in statistically significant and highly modulated (ANOVA *p* < 0.05 + Fold Change ≥ 1.5) in the specific pairwise comparisons with respect to the control, such as P vs. control and F3 vs. control (Fig. 8A and Supplementary Table S4), or high salt + P vs. high salt and high salt + F3 vs. high salt, in the case of salinity stress (Fig. 8B and Supplementary Table S5).

The employment of the two biostimulants on lettuce grown under normal condition highly modulated secondary metabolites and cofactors biosynthesis (Fig. 8A and Supplementary Table S4). Specifically, P biostimulant reported a differential modulation trend in the biosynthesis of macromolecules, such as amino acids, nucleotides, and lipids, compared to its fraction F3 (Fig. 8A and Supplementary Table S4). Considering secondary metabolites affected by biostimulants modulation, an overall down-modulation on phenylpropanoids and up-modulation on terpenoids biosynthesis have been reported. Moreover, N-containing compounds were differentially produced under P (down) and F3 (up) biostimulants. Specifically, the F3 fraction up-modulated the accumulation of aliphatic glucosinolates, alkaloids, and terpenoid alkaloids (Supplementary Table S3). Regarding the differential modulation of macromolecules by biostimulants application, F3 positively modulated the biosynthesis of fatty acids and lipids, and negatively modulated the biosynthesis of cell-structures components, suggesting an assessment of metabolism in development and energy versus fortification and defense. Specifically, F3 up-modulated the synthesis of di- and tri-acylglycerols, phospholipids, sphingolipids, and sterols.

The employment of biostimulants on lettuce under normal conditions positively modulated the hormonal profiles, reporting up modulation precursors for the synthesis of brassinosteroids (i.e., campestanol) and serotonin and melatonin (i.e., N-acetyl-serotonin).

The biosynthetic modulation effect of P biostimulant and its fraction F3 under salinity stress conditions resulted in a significant impact on secondary metabolites, hormones, cofactors, carriers, and vitamines pathways, as well as cell structures, membrane lipids, carbohydrates, and amino acids (Fig. 8B and Supplementary Table S5). Specifically, F3 positively modulated secondary metabolites biosynthesis, including N-containing compounds and phenylpropanoids, and down-modulation of terpenoids. Belong to N-containing compounds, the F3 modulated the increase of glucosinolates biosynthesis starting from hexahomomethionine, and α-solanine and α-chaconine. Moreover, F3 positively modulated flavonoid biosynthesis, including flavonoids di-C- glucosylation, luteolin, and flavonols. While anthocyanins were strictly down-modulated. The P biostimulant up-modulated terpenoids biosyntheses, such as carotenoids, saponins, and other triterpenoids.

Under salinity stress, biostimulants highly modulated the turnover of cofactors, reporting different trends based on fractions (F3) or whole extract (P). Specifically, F3 positively modulated carrier synthesis, including acyl-carrier protein and coenzyme A. The electron carriers were enhanced by F3 application, including metabolites involved in the biosynthesis of quinol, quinone, ubiquinol-10, and phylloquinone. Moreover, NAD de novo biosynthesis and pyridine nucleotide cycling were positively modulated, as well as gamma-glutamyl cycles. The positive modulation also affected the synthesis of enzyme cofactors, including ascorbates, biotin, glutathione, thiamine, and vitamin B6. These modulations were also associated with increased cell structures of lettuce plants, as well as lipids and fatty acids.

Phytohormones profiles were modulated by both biostimulants, however, with different targets. Specifically, F3 application up-modulated trans-zeatin and other cytokinins. At the same time, brassinosteroids were specifically modulated by P biostimulants. Both of them reported a strong modulation in the biosynthesis of abscisic acids.

### Data integration of transcriptomics and metabolomics outputs

**Figure 9:**
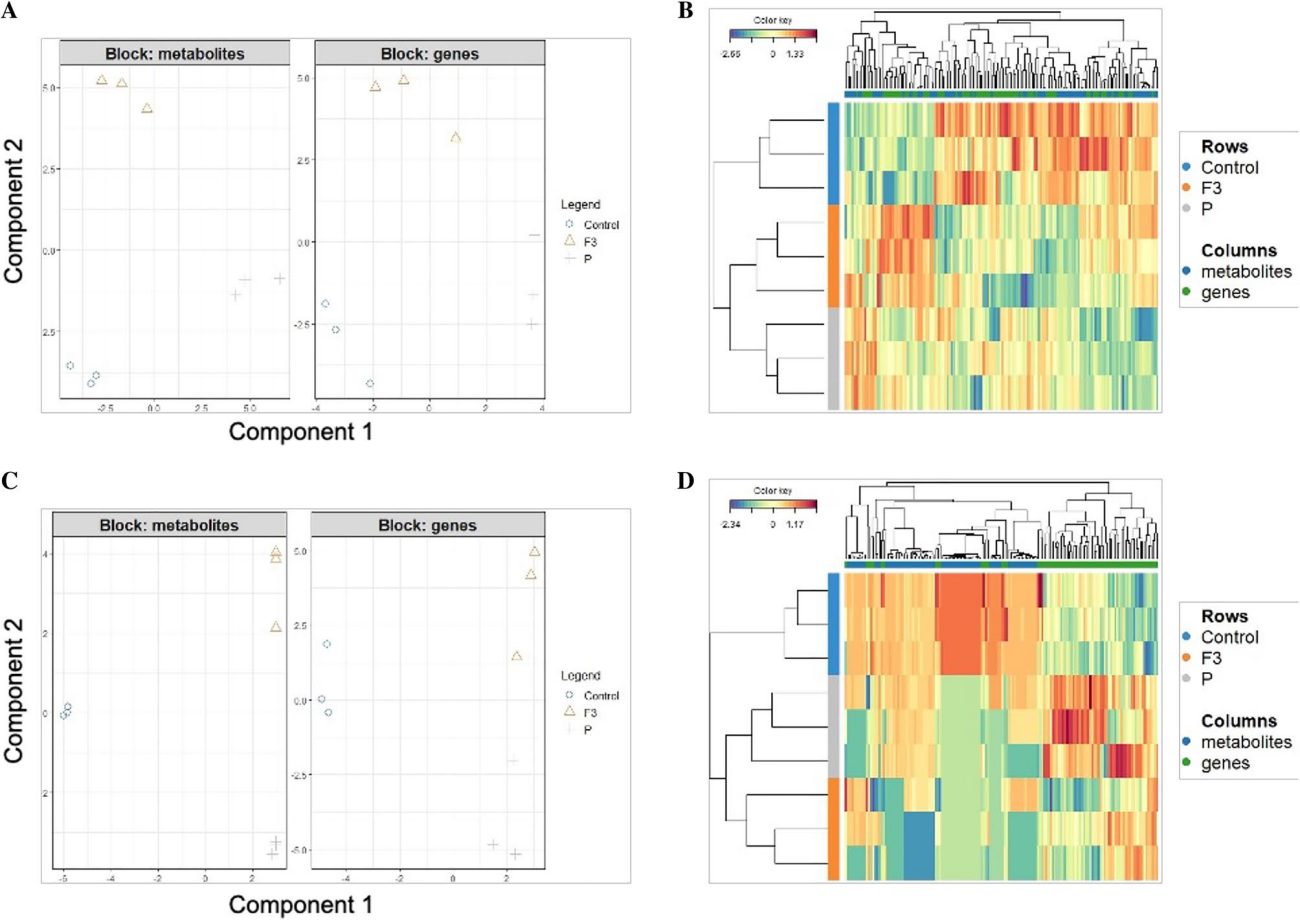
DIABLO-based data integration models for the metabolomics and transcriptomics of lettuce plants. (**A**) Block contributions for metabolomics and transcriptomics outputs discriminating among control, P, and F3 treatments in lettuce plants grown under no salt conditions. (**B**) Heatmap-based cluster jointly involving metabolomics and transcriptomics outputs in lettuce plants grown under no salt conditions. (**C**) Block contributions for metabolomics and transcriptomics outputs discriminating among control, P, and F3 treatments in lettuce plants grown under high salt conditions. (**D**) Heatmap-based cluster jointly involving metabolomics and transcriptomics outputs in lettuce plants grown under high salt conditions. Plots have been generated by using MixOmics R package (version 6.22).

A DIABLO data integration workflow was further applied to determine the joint influence of biostimulant treatments and salt stress on both the transcriptome and metabolome of lettuce plants. For this purpose, the list of DEGs and significantly different metabolites associated to each treatment were considered, and the results of the integration are displayed in Fig. 9.

#### DIABLO-based data integration under no salt conditions

In the absence of salt stress, the DIABLO model reflected a high correlation between datasets for the two components previously optimized: 0.97 and 0.96, respectively (Supplementary Fig. S6). Regarding block contributions (Fig. 9A), the same inference was observed at both metabolic and transcriptomic levels, since the first component allowed discriminating two groups, represented by the combination of control and F3 treatments apart from P treatment, whereas the second component specifically discriminated F3 treatment with respect to the control and P treatments. Overall, the joint cluster analysis indicates that both biostimulant treatments clustered apart from the control (Fig. 9B), suggesting a biostimulant-dependent effect on both transcriptome and metabolome of lettuce plants, with P treatment showing the most discriminating overall effect with respect to the control. The supervised analysis atributed to DIABLO modeling also provided the significant features (metabolites and genes) mainly responsible for the discrimination between the treatments (Supplementary Table S6).

Thus, in the case of transcriptomics, the most part of markers for both first and second components were associated to the control, being mostly represented by photosynthesis-related ontologies, such as *photosynthesis*, *photosynthetic electron transport chain*, *light reaction* (*LSAT_8X133980*, *LSAT_3X104200*, *LSAT_4X102701*, *LSAT_5X152640*, etc.), *chlorophyll binding* (*LSAT_8X133980*, *LSAT_3X104200*, *LSAT_9X104700*, *LSAT_9X92240*, *LSAT_1X48460*, etc.) and *ATP metabolic process* (*LSAT_4X39621*, *LSAT_2X93861*, *LSAT_6X26160*, *LSAT_3X84241*, etc.). Interestingly, the genes belonging to these ontologies were generally found up-regulated in the control plants, whereas they were down-regulated in both biostimulant treatments, suggesting that they induced a growth-independent response in lettuce plants. On the contrary, only some genes belonging to *chrolophyll/tetrapyrrole binding* ontologies were mostly associated with the F3 treatment (*LSAT_4X94000*, *LSAT_7X98980*, *LSAT_1X3881*, *LSAT_0X24441*, *LSAT_8X54041*, *LSAT_2X121400*) where they were up-regulated, as well as in the case of genes encoding for cytochromes P450 (*LSAT_8X58600*, *LSAT_4X114881*, *LSAT_4X123700*, *LSAT_6X113301*, *LSAT_6X86481*), thus supporting the hypothesis of the biostimulant-specific effect on lettuce. Besides photosynthesis-associated genes, the control treatment was associated with the up-regulation of several genes involved in other processes, as observed for *lyase activity* (*LSAT_6X62560*, *LSAT_9X71760*, *LSAT_9X5660*), *cellulase activity* (*LSAT_9X46241*), and *ribosome biogenesis* (*LSAT_0X13881*, *LSAT_8X99320*, *LSAT_7X108660*, *LSAT_4X76561*, *LSAT_3X35601*). In parellel, F3 also presented up-regulated biomarkers involved in lyase activity (*LSAT_2X52280*, *LSAT_8X16781*, *LSAT_2X29420*), whereas only one marker was positively associated to P treatment, related to *oxidoreductase activity* (*LSAT_8X116281*).

The metabolite markers provided by the DIABLO model depicted a proportional representation with respect to gene markers, as the most part were associated with the control and F3 treatments. Considering F3 treatment, a number of metabolites involved in the primary metabolism were positively associated with this treatment, whereas secondary metabolites were negatively associated, being in agreement with the results found at a transcriptomic level, suggesting a significant involvement of F3 on the stimulation of primary metabolism. In this sense, the F3-associated metabolites essentially included organic acid derivatives involved in amino acid biosythesis, like 3-hydroxy-3-carboxy-4,5-cyclopropylhex-5-enoate, 2-oxopent-4-enoate, and (S)-2-acetolactate, and modified amino acids, such as serinol phosphate and (indol-3-yl)acetyl-L-alanine. In contrast, some secondary metabolites were negatively associated with F3 treatment, for instance alkaloids like vinorin or 10-hydroxydihydrosanguinarine, and phenolic compounds, such as the phenolic acid derivatives 1-O-feruloyl-β-D-glucose and salicylaldehyde, and the flavonoid 7,3′-dimethylquercetin. In the case of control plants, most accumulated metabolite markers belonged to secondary metabolism, including glucosinolate derivatives (10-(methylsulfanyl)-2-oxodecanoate and (E)-1-(L-cystein-S-yl)-N-hydroxy-omega-(methylsulfanyl)heptan-1-imine), the stilbenoid pynosilvin, the lignan (-)-5′-demethylyatein, and the flavonoids leachianone G and wighteone, and the alkaloids (S)-tetrahydropalmatine and deacetylisoipecoside. Moreover, the DIABLO model spotted some lipid metabolites that were also accumulated in control plants, for instance oxidized fatty acid derivatives (i.e., (2E)-hexadecenal, 1,16-hexadecane-diol, palmitaldehyde, (9Z)-octadec-9-ene-1,18-diol), and glycerolipids, represented by 1-linoleoyl-2-palmitoleoyl-sn-glycerol 3-phosphate, 1–18:1–2-16:0-digalactosyldiacylglycerol, and dipalmitoyl phosphatidate. The combined presence of oxidized fatty acids and secondary metabolites as glucosinolates suggest the induction of a relative stress response reported for control compared with biostimulant-treated plants, which may exhibit a priming effect.

#### DIABLO-based data integration under high salt condition

Under high salt condition, both metabolomics and transcriptomics datasets presented a high correlation for the two components involved in the DIABLO analysis: 0.99 and 0.87, respectively (Supplementary Fig. S6). The modeling results of both metabolomics and transcriptomics reveal that the first component essentially distinguished both biostimulant treatments from the control, whereas the second component allowed to discriminate between the three experimental groups, especially in the case of the metabolome, whereas a partial overlapping was observed between the control and F3 treatments at a transcriptome level (Fig. 9C). In this sense, the combined cluster depicts two different subclusters, grouping the control apart from the biostimulants treatments, P and F3 (Fig. 9D). The most discriminating features responsible for this outcome show a high proportion of metabolite and transcriptomic markers associated with the control, suggesting a unique integrative response associated with these plants under salt stress in the absence of biostimulants (Supplementary Table S7).

Thus, at transcriptomic level, the majority of markers were associated with P treatment, involving the up-regulation of genes belonging a wide range of ontologies related to critical physiological processes: i) *cell wall organization* and *polysaccharide metabolic process*, mostly represented by the genes encoding for xyloglucan endotransglucosylase/hydrolases (*LSAT_3X43161*, *LSAT_3X33121*), and a probable β-D-xylosidase (*LSAT_9X108380*); ii) *carboxylic acid biosynthesis*, including genes encoding for 3-ketoacyl-CoA synthase (*LSAT_5X67800*), omega-3 fatty acid desaturase (*LSAT_2X124580*), and acetyl-coenzyme A carboxylase carboxyl transferase subunit beta (*LSAT_4X102681*), among others; iii) *structural constituent of chromatin*, mainly involving genes encoding for histones (*LSAT_8X22980*, *LSAT_1X16860*) and several transcription factors (*LSAT_7X51920*, *LSAT_9X28401*, *LSAT_5X10001*); iv) *enzyme inhibitor activity*, covering genes encoding for aspartic acid proteinase inhibitor (*LSAT_7X13760*) and a probable membrane-associated kinase regulator (*LSAT_5X148441*); v) FAD binding activity, including a gene encoding for delta(24)-sterol reductase (LSAT_8X32421); vi) an aquaporin-encoding gene (*LSAT_1X64661*) attributed to the ontology *water channel activity*; and vii) *hormone-mediated signaling pathway*, featuring up-regulated ethylene-responsive transcription factor (*LSAT_6X92940*) and an ABA receptor (*LSAT_8X13081*). In the same way, F3 induced a similar response at a transcriptome level in a much lesser extent, involving the up-regulation of two genes related to *structural constituent of chromatin* (*LSAT_6X107660*, *LSAT_8X25581*), and several genes involved in *hormone-mediated signaling pathway*, including two ethylene-responsive elements (*LSAT_3X21780*, *LSAT_8X164760*), and an ABA receptor (*LSAT_2X125581*).

Concerning the metabolome of lettuce plants under high salt conditions, the DIABLO-derived markers show a clear predominant presence of control-associated markers, mainly suggesting the induction of a stress-related response, guided by the accumulation of (-)-methyl jasmonate, the glucosinolate 2-[(2′-methylsulfanyl)butyl]maleate, as well as a reduction in the levels of gibberelin A_98_, the carotenoids zeaxanthin and zeinoxanthin, and the sterols campestanol, β-sitosterol 3-O-β-D-glucoside, 4α-hydroxymethyl-ergosta-7,24(24^1^)dien-3β-ol, and 4α-carboxy-4β,14α-dimethyl-9β,19-cyclo-5α-ergost-24(24^1^)-en-3β-ol. This stress-related response was also supported by the impairment of chlorophyll biosynthesis, represented by the accumulation of protochlorophyll a and the low-efficiency derivative dihydrogeranylgeranyl chlorophyll a, coupled with the accumulation of anthocyanins, such as delphinidin-3-O-(6″-O-malonyl)- β-glucoside-3′-O-β-glucoside, ternatin C5, and cyanidin 3-O-[2″-O-(2″′-O-(sinapoyl) xylosyl) 6″-O-(p-coumaroyl) glucoside] 5-O-[6″″-O-(malonyl) glucoside, together with other polyphenols. In contrast, regarding biostimulant treatments, the DIABLO model showed a higher number of metabolites associated with P treatment, being in accordance with the highest transcriptomic influence described earlier. Thus, P-associated metabolite markers included several brassinosteroids, represented by four different teasterone derivatives that were accumulated in P-treated plants, as well as other terpenoids (such as echinenone, *all-trans*-geranyl–geranyl monophosphate, and allopregnanolone), and alkaloids, i.e., thebaine and norcraugsodine. Overall, the accumulation of these specialized metabolites suggests that P treatment led to a resilient response of lettuce plants grown under salt stress, which was guided by a heterogenous wide-range transcriptomic outcome.

## Discussion

### Physiological parameters

Numerous studies have already highlighted how, especially for glycophytes, salinity drastically reduces production due to a combination of water, nutritional, and toxic stresses^33,41,42^. For salt-treated plants, although there was no statistically significant difference, the application of the F3 fraction increased the shoot dry weight by 6.7% compared to the control. However, for plants grown without NaCl, the use of different biostimulant fractions and the full product (P), did not lead to significant improvements. Despite Roupahel and colleagues^43^ stated that plant-derived biostimulants can trigger various mechanisms (biochemical, molecular, and physiological) aimed at improving productive performance in both suboptimal and optimal conditions, our results highlight that in this specific context (growth conditions and type of biostimulant used), the positive effects of such products were only observed under suboptimal conditions. As with other abiotic stresses, salinity from NaCl affects many aspects of biochemistry, including the photosynthetic process^44^. The previously described reduction in dry yield in salt-treated plants could thus be associated with a general physiological reorganization, with a decrease in A_CO2_, gs, and E as an adaptive defensive response. Despite several positive effects of plant-derived biostimulants on crop growth and production reported in the literature^45^, results regarding photosynthetic changes often appear contradictory^46–48^. This result is partly in line with what was observed in our experiment, where the application of fractions F2 and F3 led to a significant increase in A_CO2_, without significant alterations in gs and E parameters. Increasing the concentration of NaCl from 0 to 30 mM in the nutrient solution resulted in a fourfold increase in proline levels in plant tissues. Considering its well-known role as both ROS scavenger and osmotic regulator, proline accumulation may represent a useful strategy to mitigate the negative effects of excess NaCl^49^. However, it is important to note that under salinization conditions, all biostimulant treatments led to a significant reduction in this valuable osmolyte. In this context, biostimulants may have promoted proline catabolism, as the resulting metabolites could have provided useful energy to counteract damage induced by saline stress^50^. On the other hand, preliminary studies highlighted that, under salinity conditions, lettuce plants accumulated, beside Na^+^ and Cl^−^, a higher concentration of mineral nutrients (e.g. N, P, K and Mg)^30^. Interestingly, the increase in K concentration might be related to its role in maintaining a high K/Na cytosolic ratio, which might result correlated to the salt stress tolerance^30^. Similar to what was observed for proline, and in line with previous data^51^, the application of the P and F2 fraction significantly reduced MDA levels in plants stressed by salt. Since MDA is a specific indicator of membrane lipid peroxidation^52^, this result could indicate the activation of specific antioxidant responses to counter the oxidative burst induced by stress conditions.

### Transcriptomic analysis

#### High salt conditions

The treatment with P biostimulant on leaves of lettuce plants grown in high salinity conditions resulted in the significant up-regulation of genes involved in the *cell wall organization and biogenesis* confirming how the biostimulant could promote the plant tolerance to the stress altering the synthesis of the cell wall component, known to be a common process to prevent water loss and decrease ions transport in the plant^53^. The up-regulated genes encoded for pectinesterase, which modify the degree of methylesterification of pectins that is one of the principal plant cell walls component^54^, COBRA-like (*COBL*) protein, which have been demonstrated to be a key regulators in the cell expansion orientation and cellulose crystallinity status^55^, tracheary element differentiation-related protein, involved in the differentiation of xylematic cells characterized by the formation of a secondary cell wall^56^, glucuronoxylan 4-O-methyltrasferase and probable xyloglucan endotransglucosylase/hydrolase (*XHT*) involved in the hemicellulose modification aiming to remodelling the plant cell wall^53^, expansin, able to trigger a pH dependent cell wall relaxation enabling its expansion^57^, cellulose synthase-like protein, involved in the cellulose biosynthesis^58^ and callose synthase 7, which in *Arabidopsis* phloem is responsible for callose deposition during phloem formation and wounding recovery^59^.

The application of P also impacted the *carbohydrate catabolic process* and *polysaccharide metabolic process*, within which, the up-regulation of genes encoding for endoglucanases was observed. These enzymes play a pivotal role in the hydrolytic process of cellulose, randomly cleaving this polymer into smaller polysaccharides^60^.

Considering the BP term *organic acid biosynthesis process,* the treatment with the P biostimulant caused the up-regulation of genes possibly involved in the mechanisms underpinning the response to abiotic and biotic stresses. Indeed, the up-regulation of N-acetyl-L-glutamate synthase (*NAGS*), L-gulonolactone oxidases (*GLOase*) and a breast cancer susceptibility 1 homolog demonstrated that P treatment could activate the response against the osmotic and oxidative damage. The *NAGS* gene encodes for the first enzyme involved in the arginine biosynthesis^61^, and its over-expression in *Arabidopsis thaliana* plants caused an over accumulation of ornithine, which is an intermediate in the biosynthesis of osmoprotectant compounds, like proline and polyamines^62^. The *GLOase* gene, instead, encodes for one of the key enzymes in the ascorbic acid (AsA) biosynthesis^63^ and it is suggested to help plants tolerating salt stress^64^. In fact, the increase in the *GLOase* expression in transgenic potatoes and *Arabidopsis* led to an enhanced production of AsA, that could effectively detoxify ROS, and an improved resistance to salt stress^63,64^. In this context, the up-regulation of breast cancer susceptibility 1 homolog could play a pivotal role in the protection against oxidative damage. In fact, despite being an important tumor suppressor in animals, in plants breast cancer susceptibility gene 1 (BRCA1) can maintain the DNA structural integrity. Nevertheless, studies carried out in *A. thaliana* also demonstrate a possible role in the transcriptional regulation of intracellular ROS homeostasis under abiotic stress^65^. Furthermore, the treatment with P biostimulants in salt-stressed lettuce plants induced the up-regulation of a putative linoleate 9S-lipoxygenase that is normally involved in the regio- and stereo-specific dioxygenation of the polyunsaturated fatty acids^66^. This gene was shown to be induced by exposure to salt in *Musa paradisiaca*^67^, whilst it was suggested to be involved in the JA-elicited salt tolerance in wheat plants^68^. On the other hand, less clear is the contribution given by the induction of a nodulin-related protein 1, possibly involved in the transport of different solutes (e.g., amino acid and disaccharides)^69^,whose modulation was related to a wide range of biotic and abiotic stresses^70^, and of a 3-ketoacyl-CoA synthase (*KCS*) 19, which is involved in the biosynthesis of very long chain fatty acids. Studies of *KCS* gene family in cotton suggested that this may be involved in hormone signalling, defence and stress response^71^. Interestingly, the over-expression of *Vitis vinifera* KCS in transgenic *A. thaliana* plants conferred salt resistance, albeit the underpinning molecular mechanisms remained unclear and was ascribed to regulation of expression of ion transporters and channels, accumulation of osmotic regulating substances, and maintenance of membrane stability^72^.

Within the same BP term, the down-regulation of one gene encoding for alpha-dioxygenase (*alpha-DOX*) was also detected. The *alpha-DOX* gene are known to be involved in the biosynthesis of oxylipins, which, in turn, could be related to the generation of lipid-mediated signal in plants^73^. Interestingly, *alpha-DOX* was shown to be induced in tomato roots under different stressing conditions (both biotic and abiotic), being its modulation mediated by ABA signalling^74^.

Interestingly, P significantly impacted the *structural constituent of chromatin* and *protein dimerization activity* in plants under salinity stress. The up-regulation of seventeen genes encoding for different variants of nucleosome components (*H3.2*, *H2A.1*, *H4*, *H2B.9*, *H2B.3* and *H2A.3*) highlighted how the abiotic stress conditions may affect the nucleosome assembly/disassembly and histone variants. Histone variants differ in the amino acid sequences from the canonical ones, as previously demonstrated in *Arabidopsis*^75^. Noteworthy, changes in the histones variants, and thus in the nucleosome structure, can influence the degree of chromatin condensation, making it more or less accessible to transcription factors^76^. Abiotic stresses have already been ascertained to influence chromatin organization via epigenetic mechanisms in plants^77^. Nevertheless, these observations demonstrate that also biostimulants can trigger such mechanisms, when administered on stressed plants.

Furthermore, within the *protein dimerization activity* term, there were detected the up-regulation of one gene encoding for a desmethylxanthohumol 6-O-methyltransferase, an enzyme involved in the biosynthesis of the phenolic compound xanthohumol^78^, and two basic helix-Loop-helix (*bHLH*) transcription factors (TF), which are the most widespread TF class in plants playing an important role in abiotic stress response as well as plant growth and development ^79^. Many *bHLH* promoting salt tolerance were identified in different plant species which were able to promote different pathway such as ABA-induced salt tolerance or proline biosynthetic pathway^79^.

Further MF terms that were significantly impacted by the treatment with biostimulant P high salt conditions were *hydrolase activity, hydrolyzing O-glycosyl compounds*; *hydrolase activity, acting on glycosyl bonds* and *xyloglucan:xyloglucosyl transferase activity* within which an endoglucanase and a *XHT* were up-regulated, whilst a polygalacturonase was down-regulated. The endoglucanase genes have been shown to be involved in the hydrolysis of polysaccharides and their modulation has been observed in wheat and strawberry plants when exposed to salt stress conditions^80,81^. Interestingly, endoglucanases are enzymes playing a possible role in cell wall loosening^82^, that, together with the activity of XHT, might determine the cell wall plasticity in response to the stress^81,83^. On the other side, polygalacturonase is a hydrolytic enzyme involved in the pectin degradation and, as a consequence, in the cell wall depolymerization^84^, whose induction was shown to increase the salt-sensitivity in rice plants^85^. Beside these, other genes involved in polysaccharides synthesis, degradation and remodelling (e.g. *NFH1,* beta-fructofuranosidases, glucan endo-1,3-beta-glucosidases) were modulated by the treatment with P biostimulants. Overall, these data further strengthen our hypothesis that one of the main mechanisms triggered by P biostimulants in lettuce plants subjected to salt stress consists in cell wall biosynthesis and remodelling.

On the other hand, when compared to the P biostimulant, the F3 biostimulant fraction impacted GO terms with a lower number of genes significantly up- and down-regulated. Indeed, the F3 fraction affected the *hormone-mediated signalling pathway*, *cellular response to endogenous stimulus* and *cellular response to hormone stimulus* by inducing the up-regulation of an ethylene-responsive TF (*ERF*). *ERFs* are known to be involved in the modulation of ethylene responsive genes and to play an important role in abiotic stress response. In fact, a study reported the up-regulation of different *ERFs* genes in tomato under salinity stress highlighting their role in coping with the stress^86^. Additionally, it was detected the significant up-regulation of one gene encoding for histidine-containing phosphotransfer protein (*HPts*) 4. These are generally involved in the cytokinins signal pathway and *HPts4* in Arabidopsis has been shown to play a pivotal role in response to abiotic stress, in particular drought^87^.

Others MF terms significantly impacted by the F3 treatment in high salt conditions were *signal transducer activity*, *signalling receptor activity* and *molecular transducer activity* in which the up-regulation of HPts*4* and the down-regulation of a mitogen-activated protein kinase (*MAPK*) homolog *MMK2* gene were detected. *MAPK* cascades are key signalling pathway involved in both endogenously and exogenously stimuli and are involved in the plant immunity, growth and the response to environmental stresses^88^. Under stress conditions, salinity included, *MAPK* activation resulted also in microtubules remodelling, as in the case of *MMK2,* involved in the regulation of cytoskeleton arrangement in *Medicago sativa*^89–91^.

Although the GO enrichment analysis allowed the identification of few genes significantly affected by the treatment with the biostimulant fraction F3 in salinity stress condition, the KEGG pathway analysis provided further insight in the transcriptional responses of lettuce plants. As mentioned above, *MAPK signalling pathway* and *plant hormone signal transduction*, in fact, resulted to be significantly affected by the fraction treatment in high salt condition. These two pathways are strongly connected to each other since *MAPK* cascades are regulated by and regulate plant hormones, especially in stress conditions^92^. Abiotic stress condition induces the ethylene production which activated, as observed in this study, the up-regulation of several genes encoding for proteins involved in both *MAPK* cascade signalling and hormone signal transduction. In particular, in salt-stressed plants and treated with biostimulants fraction F3, the up-regulation of ethylene membrane-associated receptors (*ETR* and *ETR/ERS*) and ethylene response factor 1 (*ERF1*), which belong to the APETALA2-domain-containing TF and bind the promoters of different defence related genes^93^, was observed. Moreover, within the same pathway, it was detected the up-regulation of two F-box proteins, *EBF1* and *EBF2*, which, in absence of ethylene perception, target the TF *EIN3* and *EIL3*, directing them the 26S proteosome-mediated degradation^94^. Overall, these data highlight a complex regulation of ethylene signalling pathway in response to salt stress and to the treatment with biostimulant fraction F3.

Interestingly, within the *MAPK signalling pathway,* the down-regulation of genes encoding for *MAPK4* and *MAPK6,* which are known to be actively involved in the salt stress response through the MEKK1-MKK2-MAPK4/6 pathway, was observed^95^. However, the down-regulation of these genes could be related to the mode of action of the biostimulant fraction F3 in salt stress condition, since the salinity stress alone is demonstrated to induce their up-regulation^95^.

Furthermore, in salt stress conditions many metabolic processes involved in the stress-tolerance response are activated and mediated by the plant hormone abscisic acid (ABA)^96^.

Within the phosphoproteins cascade triggered by ABA, in response to biostimulant fraction F3, it was identified the up-regulation of the pyrabactin resistance/pyrabactin resistance-like (*PYR/PYL*) receptors that, in the presence of ABA, inhibit protein phosphatases 2C (*PP2C*). The inhibition of PP2C allows the activation of *SNF1*-related protein kinases 2 (*SnRK2*), that induce the transcription of ABA-responsive genes^97^. Indeed, it was shown that the overexpression of PYR/PYL in wheat confers drought tolerance, showing a connection between PYR/PYL and drought resistance^98^. More recently, the increased expression levels of PYR/PYL were shown to confer higher tolerance to drought and salt stresses and to promotes the expression of stress‐responsive gene in soybean^99^.

Focusing on the *plant hormone signal transduction* KEGG pathway, it resulted significantly impacted by both biostimulant P and its fraction F3 treatment in high salt conditions. In fact, both the treatments induced the up-regulation of genes involved in the ethylene and ABA signalling pathway, as also previously discussed, which are strongly involved in the osmotic stress response. However, within the ABA signalling pathway, the ABA-responsive element (ABRE)-binding factor (*ABF*) was down-regulated. This specific result was in contrast with previous observation regarding the modulation of ABA in high salinity conditions^100^, even though, overall, the biosynthetic and signalling pathway resulted up-regulated. Similarly, only P induced the down-regulation of *SIMKK* which is one of the *MAPKs* involved in the ethylene signalling pathway^101^.

Concerning the auxin signalling pathway, both the treatments induced the up-regulation of genes encoding for enzymes involved in it. Many studies demonstrated that under salt stress conditions plants have reduced auxin levels and decreased expression of auxin transporter^102–104^. However, it has also been demonstrated that modulation of the auxin gradients within plants plays a crucial role in the adaptive response to salt stress^105^. In particular, the activation of auxin signalling pathways, through different effectors mentioned above, can mediate many adaptive responses (i.e., activation/repression of auxin responsive genes) recruiting DNA-binding transcription factors (AUXIN RESPONSE FACTORS—ARFs)^106^. Consistently, Thus, the up-regulation of these genes confirmed the auxin-like activity exerted by vegetal-derived PHs promoting the coping with the stress^107–109^.

Similarly, both treatments induced the up-regulation of genes involved in the cytokinins signalling pathway, in particular histidine phosphor-transfer proteins (*AHP*) and response regulators A (*A-ARR*). The AHP was shown to participate in the modulation of stress responsive genes in *A. thaliana* and rice^110^. Experiments carried out in *Arabidopsis* showed that, upon salt stress, *AHP2*, *AHP3* and *AHP5* were down-regulated, demonstrating that the modulation of cytokinin signalling pathway plays a pivotal role in the response to salinity^110^. Consistently, in *Arabidopsis*, the ARR proteins mediate plant tolerance to salt stress by regulating the expression of *AtHKT1;1* that is responsible for the translocation of sodium into the xylem, thus conferring the tolerance trait^111^. In this context, the majority of type-A *ARR* were reported as up-regulated by salinity both in *Arabidopsis* and *Glycine soja*^112–114^*.*

Beside extensive similarities, the treatments with biostimulant P and its fraction F3 also showed peculiarities. Indeed, the main difference consisted in the modulation of brassinosteroids signalling pathway. In fact, the treatment with P induced the up-regulation different genes (i.e., *BSK*, *BZR1/2*, *TCH4* and *CYCD3*) encoding proteins involved at different levels in the brassinosteroids pathway, whilst the treatment with F3 fraction did not significantly modulate any of these genes. Brassinosteroids play a pivotal role in stress tolerance since they promote the cell division and elongation helping the plants to overcome the detrimental effects induced by high salinity^115^. Thus, the biostimulant P application showed positive effects on the stress coping also enhancing the brassinosteroids signalling pathway.

Focusing on the KEGG pathways significantly affected by the biostimulant P in high salt conditions, it induced the up-regulation of the majority of DEGs involved in the *carbon metabolism*, confirming the capability of the biostimulant to enhance the C metabolism in treated plants^109,116^ even in stress conditions. Similarly, P up-regulated the majority of DEGs involved in the *biosynthesis of amino acids* pathway, and also this aspect can confirm the capability of PHs biostimulant to enhance the plant stress tolerance through the production of amino acids that act as precursors of secondary metabolites and signalling molecules involved in the salt stress response. Furthermore, the up-regulation of amino acid biosynthesis was in accordance with previous study where the accumulation of the osmolyte proline was enhanced by the P treatment in lettuce^5^.

#### No salt conditions

Transcriptomic analysis of plants growth in no salt conditions treated with biostimulant P and its fraction F3 provided, instead, controversial results. Indeed, both treatments induced a general down-regulation of genes involved in the GO terms related to *photosynthesis*, *protein chromophore linkage*, *chlorophyll binding* and *electrons transport*. In fact, as further confirmed by the KEGG pathway analysis, both the treatments in no salt condition induced the down-regulation of genes encoding for photosystem II protein D1 and D2 heterodimer, which hosts cofactors responsible for the light-induced charge separation and water oxidation^117–119^, protein CP43 and CP47, composing the internal core chlorophyll *a* binding antenna^120^, and cytb559. Similarly, they down-regulated genes encoding for Photosystem I subunits PsaA, PsaB and PsaC and F-type ATPase subunits alfa, beta, a, b and c. Focusing on the photosynthesis antenna proteins, instead, only the F3 fraction significantly impacted this pathway down-regulation genes encoding for subunits of light-harvesting chlorophyll protein complex (LHC) in both high and no salt conditions.

The down-regulation of the photosynthetic system in not stressed plants following the treatments with biostimulants was coherent with the biochemical parameters recorded, albeit in contrast with previous studies, which demonstrated a positive effect of PHs on photosynthesis^28,108,121^. Other authors highlighted that PHs treatments could lead to the up-regulation of the photosynthetic machinery^122,123^, provided that samples collection was carried out few hours after treatments. In the present study, plant material was collected ten days after the last PHs treatment^30^, thereby showing long-term effects in the modulation of lettuce transcriptome, which were expected to be different respect to the results of short-term studies.

Moreover, according to the GO classification, both treatments significantly down-regulated cytochrome c oxidase subunits 2 and 3 and different subunits of NADH-ubiquinone oxidoreductase and NAD(P)H-quinone oxidoreductase involved in the oxidative phosphorylation process^124^. In contrast, only the fraction F3 in no stressed plants significantly down-regulated genes belonging to *ribosome biogenesis* and *structural constituent of ribosome*, as confirmed by the KEGG analysis pathway.

Even for genes associated with oxidative phosphorylation and ribosome, the observation made in this study did not comply with what observed in literature^125^. This most likely could be ascribable to the same reasons previously explained.

### Metabolomic profiling

Applying *Graminaceae*-derived biostimulant (P and its fraction F3) on lettuce under normal conditions produced a notable modulation of biosynthesis pathways. Notably, the overall down-modulation of phenylpropanoids and up-modulation of macromolecules in response to biostimulants could have significant implications on lettuce morphology and chemical compositions. Furthermore, N-containing compounds were differentially produced under the influence of P and F3 biostimulants. The F3 fraction promoted the accumulation of aliphatic glucosinolates, alkaloids, and terpenoid alkaloids. These compounds are known for their roles in plant defence mechanisms, suggesting that F3 may enhance the plant’s fortification against potential threats^121^. The observed down-regulation of secondary metabolism was particularly noticeable in phenylpropanoids, a class of metabolites primarily associated with the plant defence system. This finding suggests a shift in resource allocation, prioritizing growth and development, potentially leading to enhanced crop yield rather than emphasizing the plant defence mechanism^126^. Moreover, the P biostimulant showed a differential modulation trend compared to its F3 fraction. The biosynthesis of amino acids, nucleotides, and lipids was significantly increased after the F3 application, confirming its potential in regulating the production of essential cell biomolecules associated with cell development processes. This effect could be associated with the enrichment in the F3 fractions of small molecules acting as hormone-like molecules able to improve cell functions, including amino acids, lipids, and polyphenols, as well as nutrient acquisition improvement^127^. Hormones are pivotal in coordinating various physiological processes in plants. The application of biostimulants positively influenced the hormonal profiles in lettuce, leading to the up-modulation of precursors for synthesizing brassinosteroids, serotonin, and melatonin. These hormones have been linked to vital growth regulatory functions and stress responses, indicating that biostimulants may play a role in enhancing overall plant health and resilience. Specifically, brassinosteroids are important plant hormones involved in regulating growth and development^128^, while melatonin and serotonin are well-known in the mediation of physiological activities in plants, including growth development and also stress defence^129^.

Research has demonstrated that salt stress significantly impacts plant growth and development, affecting physiological and metabolic processes^42^. In our study, P and its F3 fraction differentially modulated lettuce metabolic pathways under salinity stress, highlighting the potential effect of the F3 fraction on boosting secondary metabolites biosynthesis, cell structures, and cofactors. The N-containing compounds, phenylpropanoids, and polyketides were the classes of secondary metabolites positively affected by the F3, confirmed to be involved in the plant response to different abiotic stresses^130^. Specifically,^131^ claimed that the stress response is directly correlated with aliphatic glucosinolates variation and changes in metabolites involved in physiological processes, such as photosynthesis, oxidative stress, energy, and hormone metabolism. Accordingly, the aliphatic glucosinolates accumulation was associated with the enrichment of flavonoid biosynthesis and enzyme cofactors, including ascorbates, biotin, glutathione, thiamine, and vitamin B6, known as both direct or indirect molecules involved in coping with oxidative stress caused by the accumulation of Na^+^^132^. Moreover, F3 improved the accumulation of electron carriers on lettuce affected by salinity stress, including phylloquinone, suggesting its contribution to improving the efficiency of photosystem I^133^, and the ubiquinol-10, implicated in the aerobic respiratory chain and participating in the biosynthesis and metabolism of important chemical compounds including antioxidants metabolites^134^. Furthermore, both biostimulants modulated phytohormones profiles, especially modulating the accumulation of abscisic acids to cope with salinity stress^135^. The F3 fraction specifically increased cytokinins biosynthesis, such as trans-zeatin, in response to salinity stress^136,137^.

### Multi-omics data integration to outline biological and functional bioactivities of biostimulants

The joint analysis of transcriptomic and metabolomic data in lettuce plants grown in stressing conditions and treated with either the biostimulant P or its molecular fraction F3 highlighted an induction of the hormonal response. In particular, the up-regulation of genes involved in the ABA response was detected and this was also mirrored by the increase in the level of metabolites playing a role in ABA biosynthesis, clearly depicting the modulation of ABA homeostasis for salt stress tolerance as a mechanism triggered by the biostimulants under investigation. Moreover, at transcriptomic level, the induction of auxin responsive genes and the modulation of both cytokinin and brassinosteroids have also been observed. Despite common effects, the biostimulant P and its molecular fraction F3 also displayed the modulation of peculiar mechanisms in lettuce plants exposed to high salinity. The F3 fraction was shown to regulate the activity of transcription factors involved in ethylene response and the down-regulation of effectors belonging to the MAPK signaling pathway, that are generally up-regulated by salt stress. Interestingly, this observation might unravel a novel mode of action of plant-based PH to alleviate the consequences of salt stress in plants. This mechanism could be further assisted by the concomitant over-accumulation of secondary metabolites (e.g. N-containing metabolites and phenylpropanoids), already well-described as key players in protecting plants from the molecular consequences of abiotic stresses in general. At metabolomic level, the treatment with P biostimulant induced a down-accumulation of secondary metabolites, possibly suggesting a reduction in the ROS scavenging capacity of lettuce plants. Nevertheless, the up-regulation of specific genes (i.e., *NAGS* and *GLOase*) that lead to the synthesis of osmoprotectants (e.g., ornithine) and antioxidants (i.e., Ascorbic Acid) clearly showed the role played by specific metabolites in the response to salt stress in P-treated lettuce plants. Consistently with the modulation of auxin response, salt-stressed lettuce plants treated with biostimulant P showed an increase in molecular activities related to the biogenesis and the plasticity of the cell wall (e.g., pectinesterases, xyloglucan endotransglucosylase/hydrolases, expansins and cellulose synthase-like proteins), further confirming the modulation of this cell organ as a mechanism to counteract osmotic stress. Interestingly, this process might be synergistic with the increased synthesis of fatty acids and lipids, that might be required to modulated cell membranes. It is worth noticing that salt-exposed plants and treated with P biostimulant displayed an induction of genes encoding for histones variants, which can alter the structure of nucleosomes and thus affect the chromatin condensation. Although abiotic stresses and microbial biostimulants have already been demonstrated to modulate gene expression via epigenetics, to the best of our knowledge, this is the first time that such mechanism is discovered for protein hydrolysates.

On the other hand, when considering plants grown in not stressing conditions and treated with either the biostimulant P or its fraction F3, the analyses of physiological, metabolomic and transcriptomic data are just partially in agreement. In fact, whilst the down-accumulation of secondary metabolites and the modulation of specific hormone pathways might suggest a preferential allocation of resources towards growth, we observed a down-regulation of the photosynthetic machinery that is coherent with no significant variation in the CO_2_ assimilation rate and in the leaves biomass. Nevertheless, considering the accumulation of aliphatic glucosinolates, alkaloids, and terpenoid alkaloids induced by F3 treatment, a priming effect against biotic stress cannot be ruled out.

## Conclusions

It is very clear that, in the agricultural sector, meeting the challenge of climate change and its effects, thereby including the increasing incidence of the salinity problem in cultivated soils, urgently requires the development of specific agronomic practices and the identification of appropriate tools. With regard to the latter, the availability of biostimulant preparations that promote crop resilience to stresses, salinity among them, is of particular interest. The evidence provided within the present research highlighted that, despite triggering either common or peculiar mechanisms, the P biostimulants and its fraction F3 could lead to similar outcomes when considering the accumulation of protective osmolytes and the tolerance to oxidative stress, albeit F3 fraction had a slightly higher growth promotion effects in high salt conditions. Overall, the data here presented demonstrates that, although plant biostimulants are accounted as effectors able to improve plant fitness acting at different levels (biochemical, molecular, and physiological), their mode of action can be strongly dependent on i) the plant physiological status and ii) their composition (i.e., non-fractioned biostimulants vs. fractioned ones). This latter aspect further underscores the need of deeply investigating the bioactivity of the different molecular components of a biostimulants to fully exploit their beneficial potential considering different plant species and different environmental/edaphic conditions, in a context not only of circular economy but also of precision agriculture.

## Material and methods

### Experimental design and biostimulant treatments

The experiment was carried out at the Department of Agriculture of the Federico II University of Naples, in an unheated greenhouse with passive ventilation (Portici, Italy; 40°48′ N, 14°20′ E, 29 m.a.s.l.) during the spring season 2022. At the phenological stage of three true leaves, lettuce plants (*Lactuca sativa* L.) cv. ‘Maravilla De Verano Canasta’ (provided by Pagano Domenico e Figli, Scafati, Salerno, Italy) were transplanted into pots (14 cm diameter and 1.6 L total volume) filled with a mixture (v/v) of 10% perlite and 90% 3-mm quartz sand at a density of 14 plants m^–2^. After transplant, lettuce plants have been exposed to two levels of salinity stress (No Salt—0 mM NaCl and High Salt—30 mM NaCl) and treated with a protein hydrolysate biostimulant and its molecular fractions. Specifically, the biostimulants adopted for the study consisted in a *Graminaceae*-derived protein hydrolysate (hereafter referred to as P), along with its three molecular fractions F1 (> 10 kDa), F2 (1–10 kDa), and F3 (< 1 kDa). The fractions were appropriately diluted to ensure an equivalent N supplementation to all the plants. All treatments occurred via foliar spray, started on the tenth day after transplantation (DAT) and subsequently applied weekly. Control plants were treated with distilled water instead of protein hydrolysates. Each treatment was replicated three times (n = 3) and arranged using a completely randomized block design. In total, the experiment comprised 30 experimental units, each unit containing 5 plants. For further details on the experiment, additional information can be found in^30^. Plants were harvested at commercial maturity, i.e., 44 days after transplant, and samples were either dried or frozen in liquid N for subsequent analyses.

### Analysis of physiological parameters

At harvest, two plants were selected from each experimental unit and of these, eight leaves were sampled. The leaf samples were immediately frozen in liquid nitrogen and subsequently stored at − 80 °C for future analyses. The remaining three plants from each experimental unit were harvested and dried in a ventilated oven to determine the shoot dry weight. Between 11:00 and 13:00 on the day before harvesting, gas exchange measurements [net assimilation rate (A_CO2_; μmol CO_2_ m^–2^ s^–1^), stomatal conductance (gs; mmol H_2_O m^–2^ s^–1^), and transpiration (E; mol H_2_O m^–2^ s^–1^)] were taken on five healthy young leaves per experimental unit using a compact photosynthesis system LCi T (ADC Bioscientific Ltd., Herts, UK). Instantaneous water use efficiency (WUEi; μmol CO_2_ mol^–1^ H_2_O) was calculated as the ratio of A_CO2_ to E. Proline and malondialdehyde (MDA) were measured in leaves by using the methods described previously^138,139^, respectively. Briefly, for the determination of proline content 0.5 g of lettuce leaves were homogenized in 3% aqueous sulfosalicylic acid and then filtered with Whatman filter paper. The filtrate was reacted with acid-ninhydrin and glacial acetic acid for 1 h at 100 °C and the reaction mixture was extracted with toluene. MDA content, instead, was determined homogenizing 0.25 g of lettuce leaves with 0.1% trichloroacetic acid (TCA). The homogenate was centrifuged at 10,000 g for 5 min and an aliquot of supernatant was mixed with 20% TCA and 0.5% thiobarbituric acid (TBA). The mixture was incubated at 95 °C for 30 min and then centrifuged at 10000 g for 10 min. The absorbance of the supernatant was measured at 532 nm.

### RNA extraction, quantification and qualification

Total RNA was extracted from frozen lettuce leaves using Spectrum Plant Total RNA Kit (Sigma-Aldrich, St. Louis, MO, USA) following the manufacturer’s instructions. RNA integrity was assessed using the RNA Nano 6000 Assay Kit of the Bioanalyzer 2100 system (Agilent Technologies, CA, USA). mRNA was purified from total RNA using poly-T magnetic beads, followed by fragmentation carried out with divalent cations under elevated temperature. First strand cDNA was synthesized using random hexamer primer and M-MuLV Reverse Transcriptase (RNase H^−^), whereas second strand cDNA was synthesized by using DNA Polymerase I and RNase H. Blunt ends were obtained via exonuclease/polymerase activities. The 3′ ends of DNA fragments were adenylated and adaptor with hairpin loop structure were ligated. Library fragments were further purified through AMPure XP system (Beckman Coulter, Beverly, USA), to enrich the fraction with length ranging between 370 and 420 bp. PCR was then carried out with Phusion High-Fidelity DNA polymerase, Universal PCR primers and Indexed Primer. At last, PCR products were purified (AMPure XP system) and library quality was assessed on the Agilent Bioanalyzer 2100 system.

The clustering of the index-coded samples was performed on a cBot Cluster Generation System using TruSeq PE Cluster Kit v3-cBot-HS (Illumina) according to the manufacturer’s instructions. After cluster generation, the libraries were sequenced on an Illumina Novaseq platform and 150 bp paired-end reads were generated. Raw data were cleaned by removing reads containing adapter, poly-N and low-quality sequences. All the downstream analyses were based on clean, high-quality data.

Reference genome and gene model annotation files were downloaded from the genome website directly (http://ftp.ebi.ac.uk/ensemblgenomes/pub/release-54/plants/fasta/lactuca_sativa/dna/Lactuca_sativa.Lsat_Salinas_v7.dna_sm.toplevel.fa.gz). Hisat2 (v. 2.0.5) was exploited to build the reference genome and to align paired-end clean reads. The quantification of gene expression level was carried out using featureCounts (v. 1.5.0-p3), whereas Fragments Per Kilobase of transcript sequence per Millions base pairs sequenced (FPKM) of each gene was calculated based on the length of the gene and reads count mapped to this gene. The differential expression between treated and control plants (three biological replicates per condition) was calculated using the DESeq2 R package (v. 1.20.0). The resulting P-values were adjusted using Benjamini and Hochberg’s approach for false discovery rate. Gene Ontology (GO) enrichment analysis of differentially expressed genes was carried out by exploiting clusterProfiler R package (v. 3.8.1); GO terms with corrected *P*-value lower than 0.05 were considered significantly enriched by differentially expressed genes. The clusterProfiler R package (v. 3.8.1) was also used to test the statistical enrichment of differential expression genes in KEGG pathways^37–39^.

### UHPLC-QTOF-MS metabolomics analysis

The untargeted metabolomics analysis was conducted as follows: extraction was carried out using 80% aqueous methanol with a homogenizer (Polytron PT 1200 E, Kinematica AG, Switzerland), followed by centrifugation (15 min, 6,000 × g; Eppendorf 5810R, Hamburg, Germany) and filtration through a cellulose membrane (0.22 µm), as previously described^42^. Subsequently, ultra-high-pressure liquid chromatography coupled to quadrupole-time-of-flight mass spectrometry (UHPLC/QTOF-MS; 6550 iFunnel from Agilent Technologies, Santa Clara, CA, USA) was used for the analysis following previously optimized parameters^42^. The features were aligned with an accuracy of 5 ppm and a retention time shift of 0.05 min. Annotation of the features was accomplished using the “find-by-formula” algorithm with the Agilent Profinder B.07 software. The isotopic pattern of molecular features, including accurate monoisotopic mass, isotope spacing, and ratio, was used for annotation, following the method previously described^42^. The PlantCyc 15.1.1 database (Plant Metabolic Network) was utilized to identify compounds. The annotation approach corresponded to a Level 2 of confidence, adhering to the COSMOS initiative for standardization in metabolomics^140^.

The raw data acquired from the instruments were then processed using (Log2 normalized, 75 percentiles filtered, and median baselined) Mass Profiler Professional B.14 (Agilent Technologies, Santa Clara, CA, USA). Using the same software, a fold-change heatmap for naïvely describing the hierarchical similarity across treatments according to cluster analysis (HCA, Euclidean distance—Ward’s linkage rule). To identify differential metabolites, Volcano Plot analysis was done in Mass Profiler Professional. Therein, analysis of variance (ANOVA; *p* < 0.05, Benjamin multiple testing correction) followed by Fold-change analysis (FC ≥ 1.5). Differential metabolites were finally interpreted in the Omic Viewer Pathway Tool of PlantCyc (Stanford, CA, USA; Access: July 2023) for pathway analysis^141^.

### Multi-omics integration of metabolomics and transcriptomics outputs

The datasets obtained from the Volcano analyses of both metabolomics and transcriptomics approaches were jointly analyzed applying the Data Integration Analysis for Biomarker discovery using Latent variable approaches for Omics studies (DIABLO) framework core as part of the “mixOmics” R package (v. 6.22). One model was applied to each experimental condition (no salt and high salt), involving the three treatments showing the most significant outcomes from their individual approaches: control, P, and F3. Firstly, a sparse partial least squares model (sPLS) was employed to determine the correlation between both datasets. The DIABLO model was further subjected to optimization by applying the tuning function of the framework, which calculates the optimal number of components with the associated lowest overall balanced error rate considering centroids distances. In this case, the optimized model was built on the basis of two components. The results for the optimized DIABLO model, based on sPLS multiblock discriminant analysis, were represented through the block contribution plots for each dataset (“metabolites” for metabolomics and “genes” for transcriptomics approaches). Finally, an integrative cluster was provided to get insight into the combined influence of treatments on both metabolomic and transcriptomic outputs. The features showing the highest combined influence into the discrimination between treatments for each dataset were calculated for each component and identified as significant markers.

### Statistical analysis

Morpho-physiological and biochemical parameters were analyzed with the SPSS 28 software package (IBM, Armonk, NY, USA) and are presented as mean ± standard error, n = 3. The mean effects were subjected to two-way ANOVA (Salt level × biostimulant). A *t*-test was employed to compare the mean effect of salt, and Tukey’s HSD test was performed to separate both the biostimulant mean effect and salinity × biostimulant interaction. All tests were deemed significant at *p* = 0.05.

Concerning metabolomics, the dataset was subjected to alignment, normalization at the 75th percentile, log2 transformation, and baselined to the median abundance of all samples before their analysis using Mass Profiler Professional (v.15.1, Agilent®). The multivariate chemometrics analysis consisted of an unsupervised hierarchical cluster analysis (HCA) derived from a fold change-based heatmap (Euclidean distance, Ward’s linkage rule) and a supervised orthogonal projection to latent structures discriminant analysis (OPLS-DA), using the SIMCA software (v.16, Umetrics®, Malmö, Sweden). Furthermore, OPLS-DA was combined with a variable importance in projection (VIP) analysis to detect those metabolites showing the highest influence in the discrimination between treatments, the so-called VIP markers, assuming a VIP score threshold of 1.15. The quality of OPLS-DA models was further evaluated in terms of goodness-of-fit (R^2^Y) and goodness-of-prediction (Q^2^Y), being statistically validated through cross-validation analysis of variance (CV-ANOVA) and excluding the overfitting by permutation test (n = 200).The metabolomics dataset was further analyzed in terms of one-way ANOVA followed by Tukey’s post hoc test (Bonferroni multiple testing correction, *p* < 0.05) and fold change analysis (FC, cut-off =  ± 1.5) to identify the differential metabolites by the Mass Profiler Professional software. Differential metabolites were subjected to pathway analysis and interpreted through the Omics Viewer Pathway by PlantCyc (Plant Metabolic Network®, Stanford, CA, USA; available at https://pmn.plantcyc.org).

### Ethics approval and consent to participate

We declare that the plant material in the experiment was collected and studied in accordance with relevant institutional, national, and international guidelines and legislation, and all the steps were performed in accordance with the relevant guidelines and regulations.

### Supplementary Information

Supplementary Information 1.

Supplementary Table S2.

Supplementary Table S3.

Supplementary Table S4.

Supplementary Table S5.

Supplementary Table S6.

Supplementary Table S7.

## Data Availability

The authors declare that the data supporting the findings of this study are available within the paper and its Supplementary Information files. The RNA sequencing data have been deposited in the NCBI SRA database (accession no. PRJNA1021776).
