# Supplementary material for: Integrated multi-omic approach reveals the effect of a Graminaceae-derived biostimulant and its lighter fraction on salt-stressed lettuce plants
Source: Sci Rep. 2024 May 10;14:10710. doi: 10.1038/s41598-024-61576-4 (PMC11087557; doi:10.1038/s41598-024-61576-4)
Supplement: Supplementary file 1 — Supplementary Information 1. [file 41598_2024_61576_MOESM1_ESM.pdf]

Supplementary Table S1. Statistical analysis of cDNA libraries obtained from lettuce leaves subjected to different stressing conditions (High salt and No salt) and biostimulant treatments (P and F3).

Sample	Raw reads	Raw bases (G)	Clean reads	Clean bases (G)	Error rate (%)	Q20 (%)	Q30 (%)	GC content (%)	Total mapped	Unique mapped
NoSalt_ Control	47171164	7.07	46754161	7.01	0.03	96.28	90.20	44.02	43602162 (93.32%)	41646995 (89.18%)
NoSalt_P	43719009	6.56	43408143	6.51	0.03	96.46	90.58	44.12	41012869 (94.48%)	39362987 (90.69%)
NoSalt_F3	50332875	7.55	49967822	7.50	0.03	96.59	90.84	44.06	47136711 (94.33%)	45064504 (90.17%)
HighSalt_ Control	49289894	7.39	48928792	7.34	0.03	96.42	90.49	43.87	45838488 (93.73%)	44094531 (90.18%)
HighSalt_P	51150056	7.67	50741201	7.61	0.03	96.65	90.95	43.87	47961230 (94.53%)	46307456 (91.26%)
HighSalt_F3	44464926	6.67	44084239	6.61	0.03	96.64	90.95	43.37	41755170 (94.73%)	40391208 (91.64%)

Supplementary Table S2. List of genes significantly modulated by treatments with respect to untreated control plants.

Supplementary Table S3. Filtered raw data obtained through metabolomic analysis.

Supplementary Table S4. List of significant metabolites obtained from volcano analysis (one-way ANOVA $p < 0.05$ + Fold change 1.5) between treatments (P and F3) and control condition.

Supplementary Table S5. List of significant metabolites obtained from volcano analysis (one-way ANOVA $p < 0.05$ + Fold change 1.5) between treatments (P and F3) and high salt condition.

Supplementary Table S6. Loading plots and marker list for the most discriminant features among treatments associated with metabolomics and transcriptomics datasets by the component 1 and 2 under no salt condition.

Supplementary Table S7. Loading plots and marker list for the most discriminant features among treatments associated with metabolomics and transcriptomics datasets by the component 1 and 2 under high salt condition.

A B

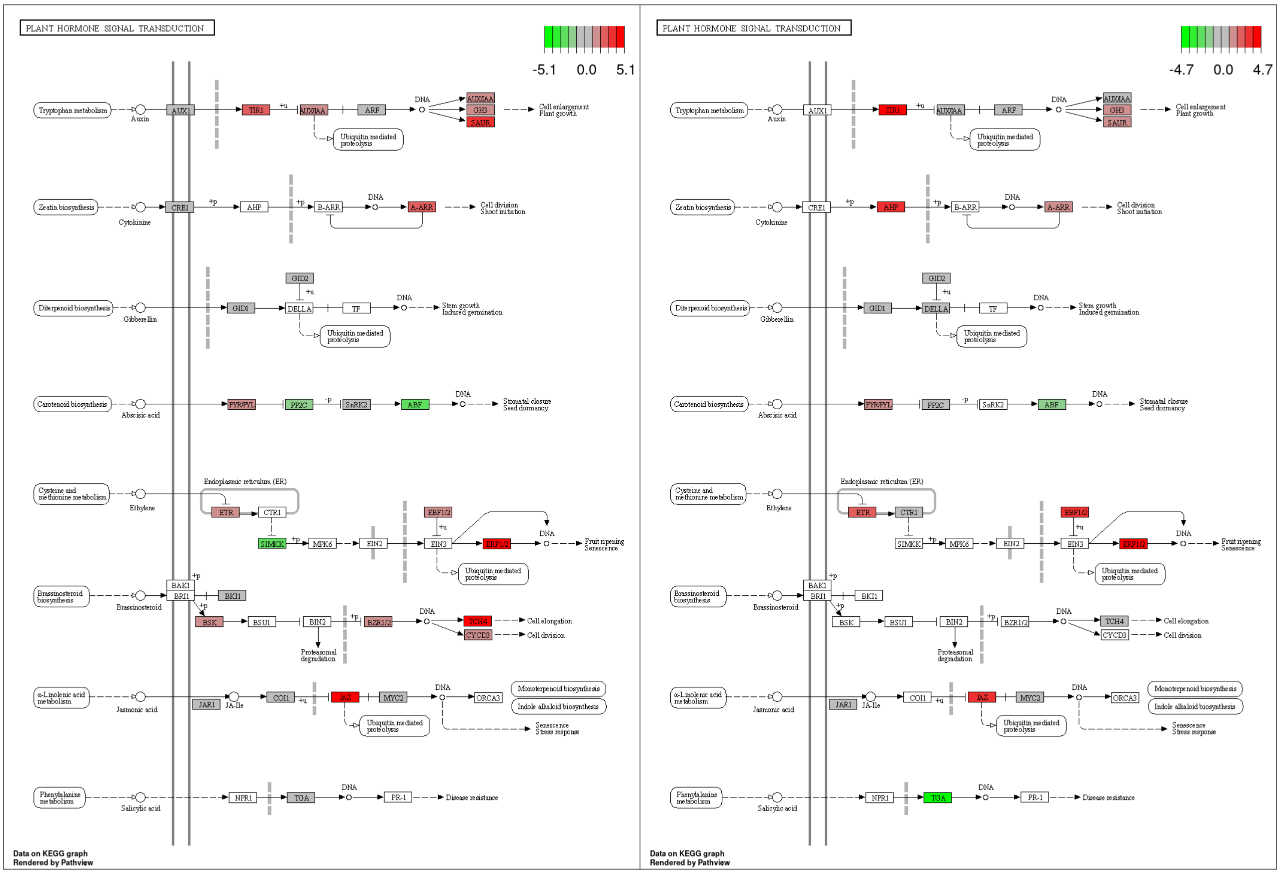

Supplementary Figure 1. Representative figure reporting the *plant hormone signal transduction* (lsv04075) pathways[1–3] as affected by P (A) and F3 (B) treatments in lettuce plants grown in high salt conditions. DEGs involved in the pathways are highlighted in red for up-regulated and green for down-regulated.

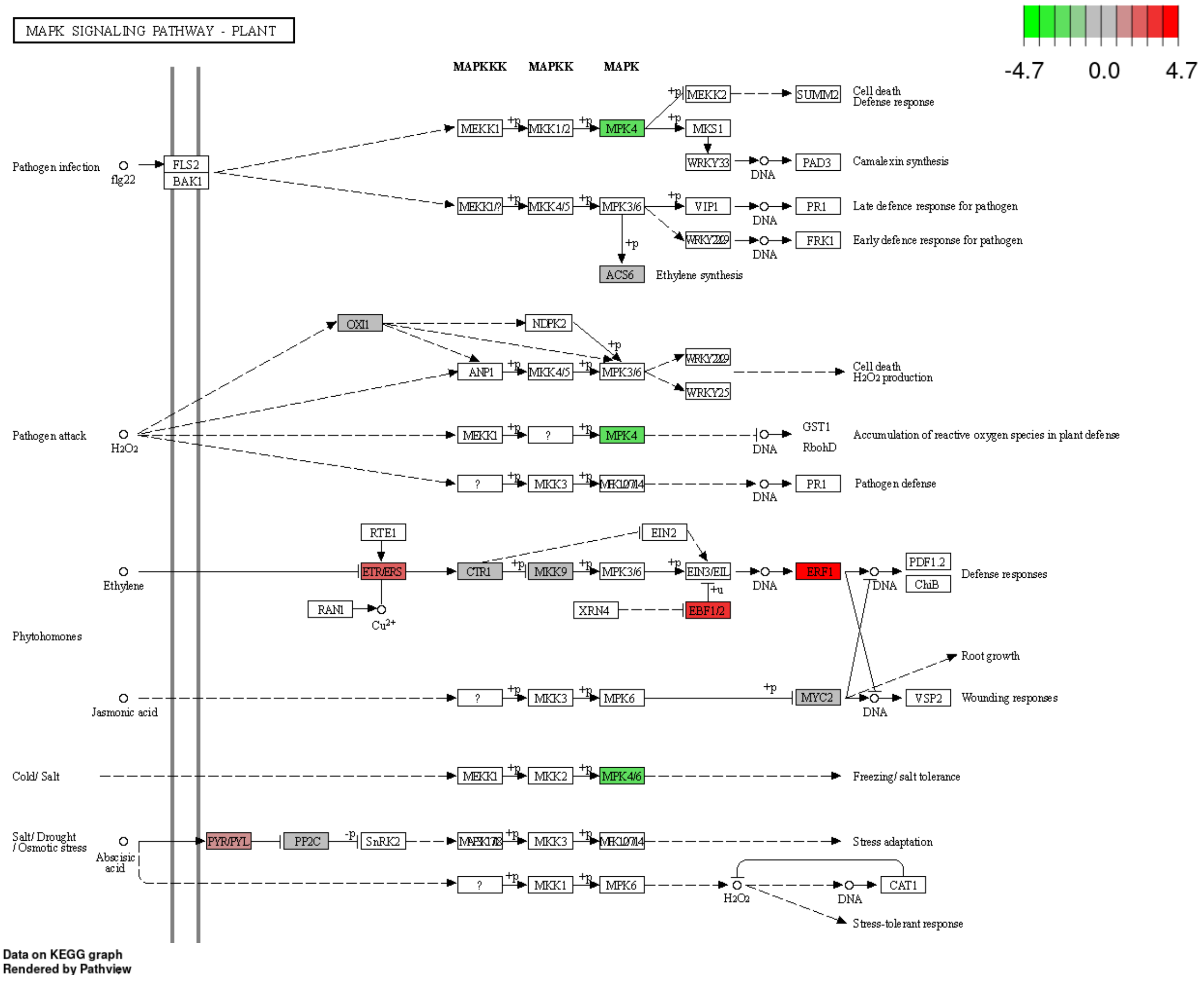

Supplementary Figure 2. Representative figure reporting the *MAPK signalling* (lsv04016) pathway[1–3] as affected by F3 treatments in lettuce plants grown in high salt conditions. DEGs involved in the pathways are highlighted in red for up-regulated and green for down-regulated.

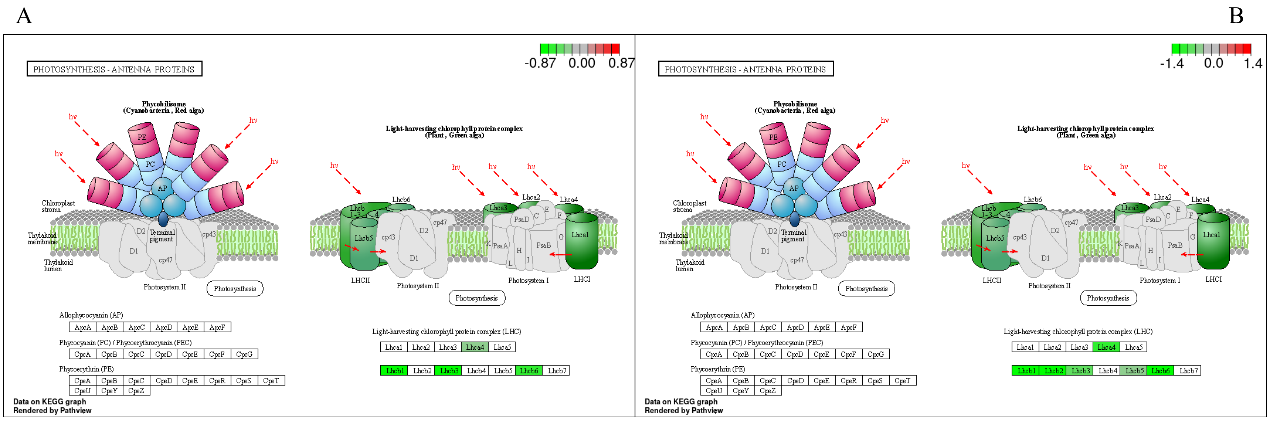

Supplementary Figure 3. Representative figure reporting the *photosynthesis -antenna proteins* (lsv00196) pathway[1–3] as affected by F3 treatment in lettuce plants grown in high salt (**A**) and no salt (**B**) conditions. DEGs involved in the pathways are highlighted in red for up-regulated and green for down-regulated.

A B

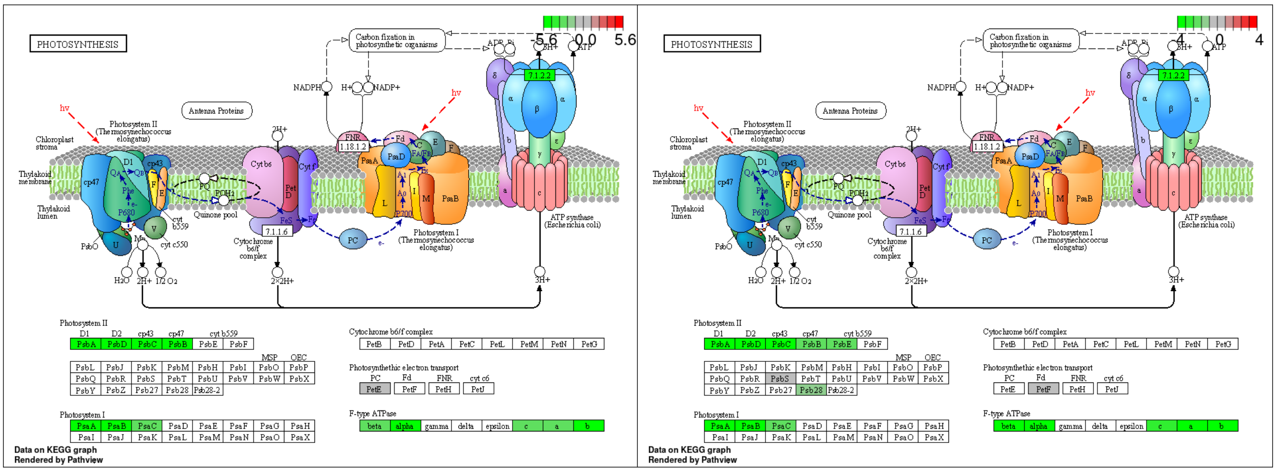

Supplementary Figure 4. Representative figure reporting the *photosynthesis* (lsv00195) pathway[1–3] as affected by P (**A**) and F3 (**B**) treatments in lettuce plants grown in no salt conditions. DEGs involved in the pathways are highlighted in red for up-regulated and green for down-regulated.

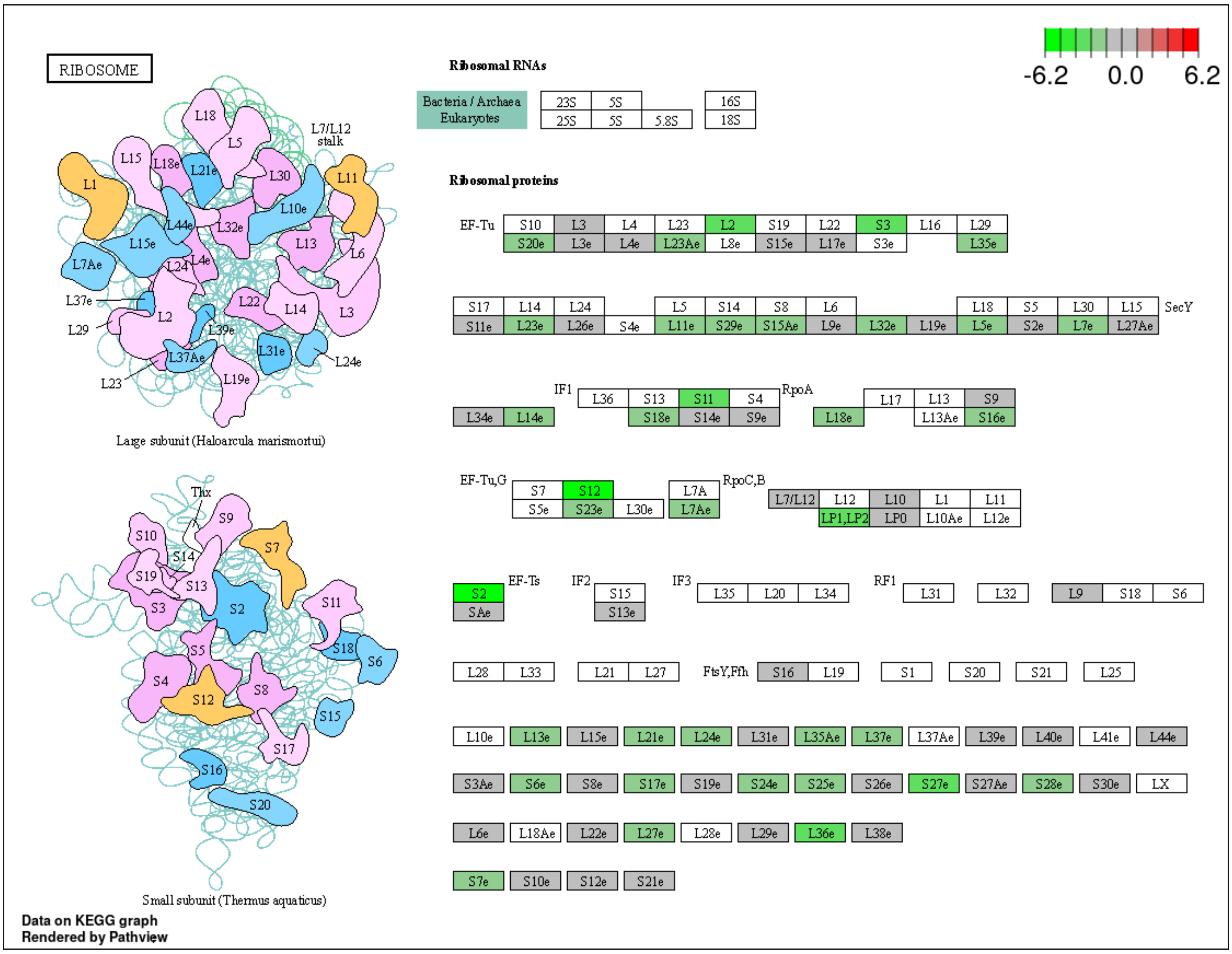

Supplementary Figure 5. Representative figure reporting the *ribosome* (lsv03010) pathway[1–3] as affected by F3 treatment in lettuce plants grown in no salt conditions. DEGs involved in the pathways are highlighted in red for up-regulated and green for down-regulated.

A

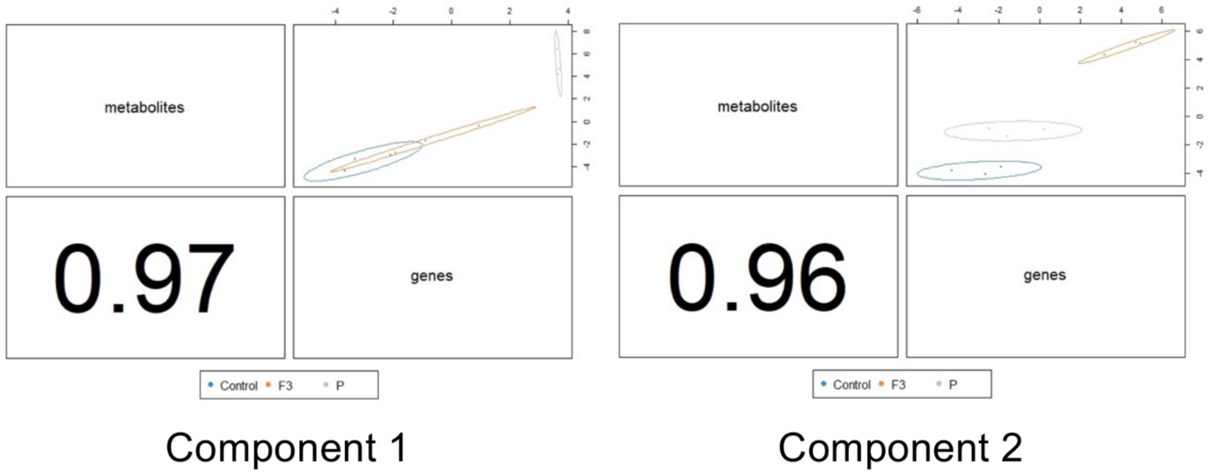

B

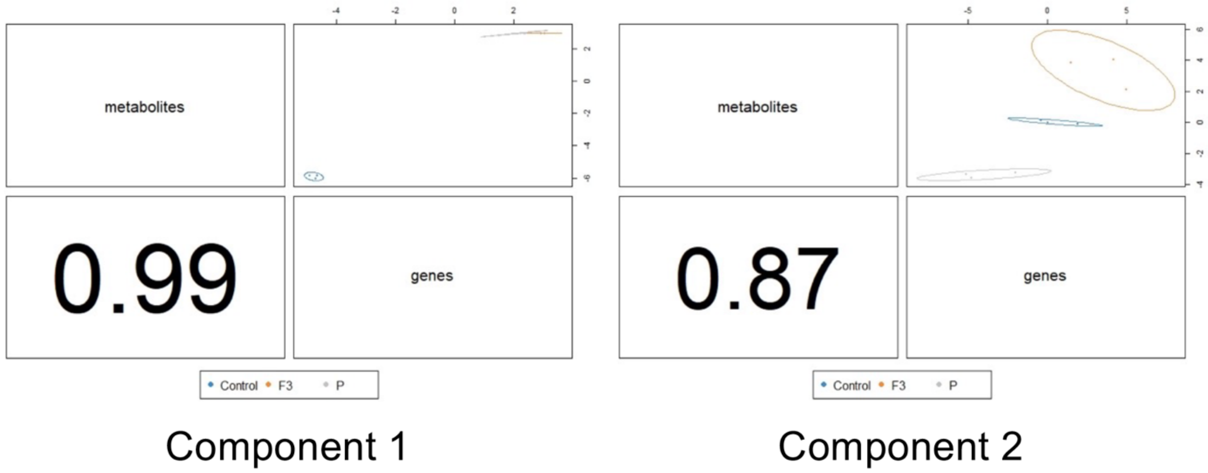

Supplementary Figure S6. Correlation matrix comparing both omics data blocks derived from the DIABLO model for each previously optimized principal component under (A) no salt and (B) high salt conditions.

Reference

1. Kanehisa, M. & Goto, S. KEGG: Kyoto Encyclopedia of Genes and Genomes. *Nucleic Acids Research* **28**, 27–30 (2000).

2. Kanehisa, M., Furumichi, M., Sato, Y., Kawashima, M. & Ishiguro-Watanabe, M. KEGG for taxonomy-based analysis of pathways and genomes. *Nucleic Acids Research* **51**, D587–D592 (2023).

3. Kanehisa, M. Toward understanding the origin and evolution of cellular organisms. *Protein Science* **28**, 1947–1951 (2019).
